# Supplementary material for: Comprehensive analysis of coding variants highlights genetic complexity in developmental and epileptic encephalopathy
Source: Nat Commun. 2019 Jun 7;10:2506. doi: 10.1038/s41467-019-10482-9 (PMC6555845; doi:10.1038/s41467-019-10482-9)
Supplement: Supplementary file 1 — Supplementary Information [file 41467_2019_10482_MOESM1_ESM.pdf]

Supplementary Information

Comprehensive analysis of coding variants highlights genetic complexity in developmental and epileptic encephalopathy

Takata et al.

Contents

Supplementary Figures 3
Supplementary Figure 1. Histogram of per-individual URV counts 3
Supplementary Figure 2. Principal component analysis 4
Supplementary Figure 3. Confirmation of sex using genotype data 5
Supplementary Figure 4. Analysis of properties of non-58EE/DEE gene dURVs among pURV carriers using transcriptome data of 53 tissue/cell types 7
Supplementary Figure 5. Analysis of enrichment of doubleton and tripleton rare variants in EE/DEE..8
Supplementary Figure 6. Quantile-quantile plot of P values in exome-wide association study of SNPs 10
Supplementary Figure 7. Power calculation with our sample size 11
Supplementary Figure 8. Comparison between EE/DEE and common epilepsies. 12
Supplementary Figure 9. Plots of results in enrichment analyses of various types of URVs in EE/DEE with or without gnomAD-based filtering. 13
Supplementary Tables 14
Supplementary Table 1. Subclassification of EE/DEE cases (743 individuals that passed QCs) 14
Supplementary Table 2. Classification of URVs 15
Supplementary Table 3. List of 58 known EE/DEE genes 16
Supplementary Table 4. Classification of pURVs in HGMD 18
Supplementary Table 5. List of recurrent pathogenic variants in 58EE/DEE genes 20
Supplementary Table 6. Detailed result of GO enrichment analysis..21
Supplementary Table 7. Clinical manifestations of DD/DEE cases with a confirmed de novo mutation in *NF1* or *CACNA1E* 22
Supplementary Notes 23
Supplementary Note 1. Detailed information of dURVs in 58EE/DEE genes in controls 23
Supplementary Note 2. Consideration on possible explanations for enrichment of dURVs in non-58EE/DEE genes in EE/DEE cases carrying pURVs in 58EE/DEE genes 24
Supplementary Note 3. Analysis of doubleton and tripleton rare variants 26
Supplementary Note 4. Exome-wide association study of SNPs..27
Supplementary Note 5. Comparison between EE/DEE and common epilepsies 27
Supplementary Note 6. Confirmation of key findings by updating ExAC to gnomAD 28
Supplementary Note 7. Consortium membership 29
Supplementary References 36

Supplementary Figures

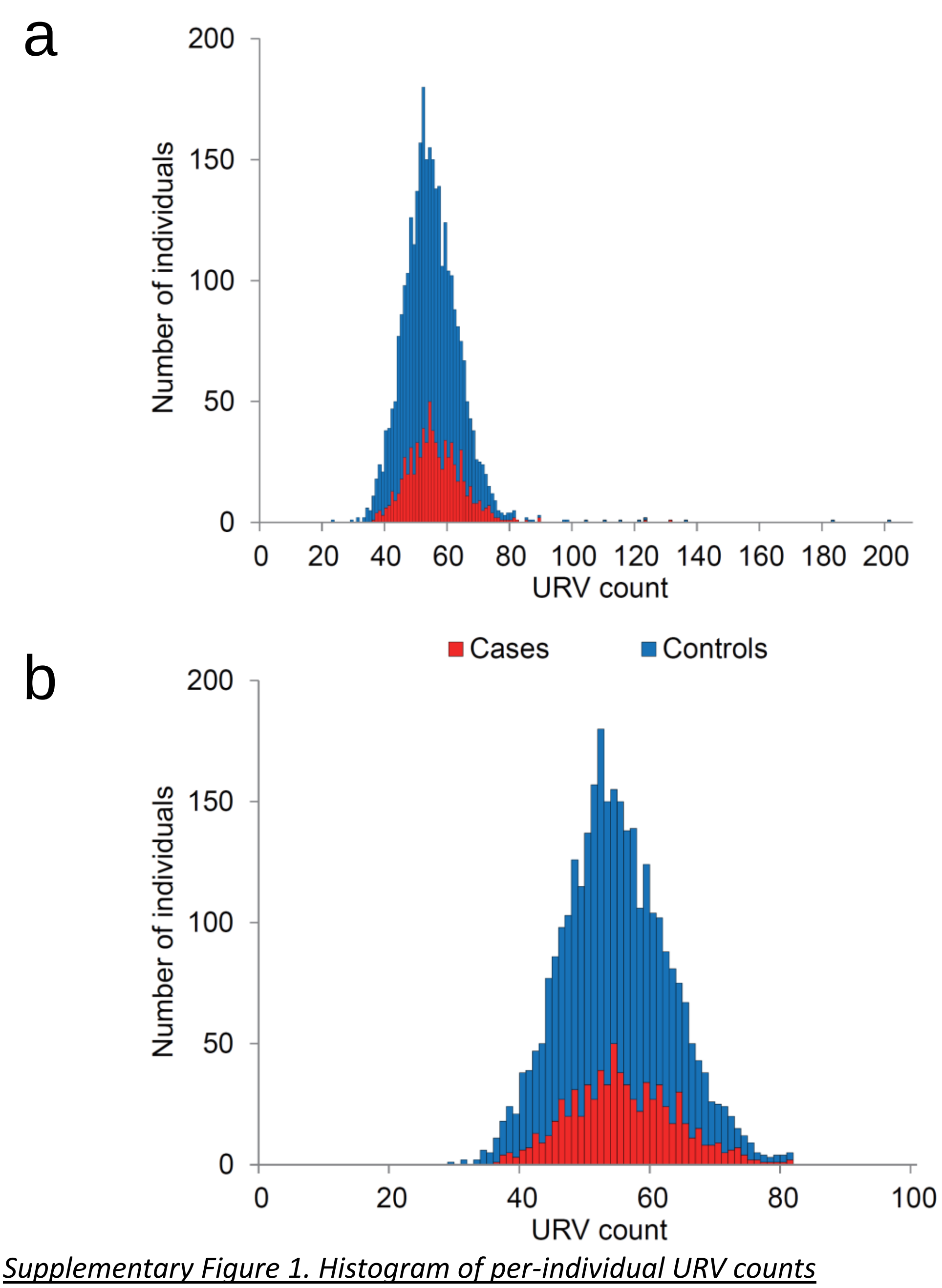

Supplementary Figure 1. Histogram of per-individual URV counts

(**a**) A histogram of per-individual URV counts (X-axis) and the numbers of individuals with the corresponding URV counts (Y-axis) in 749 EE/DEE cases (red) and 2,381 controls (blue) originally included in our analysis. Because there were some individuals with exceptionally small or large numbers of URVs, we excluded these outliers (six cases and 15 controls, Smirnov-Grubbs $P < 0.001$). (**b**) A histogram after excluding the outliers, which follows an approximately normal distribution regardless of the case-control labels.

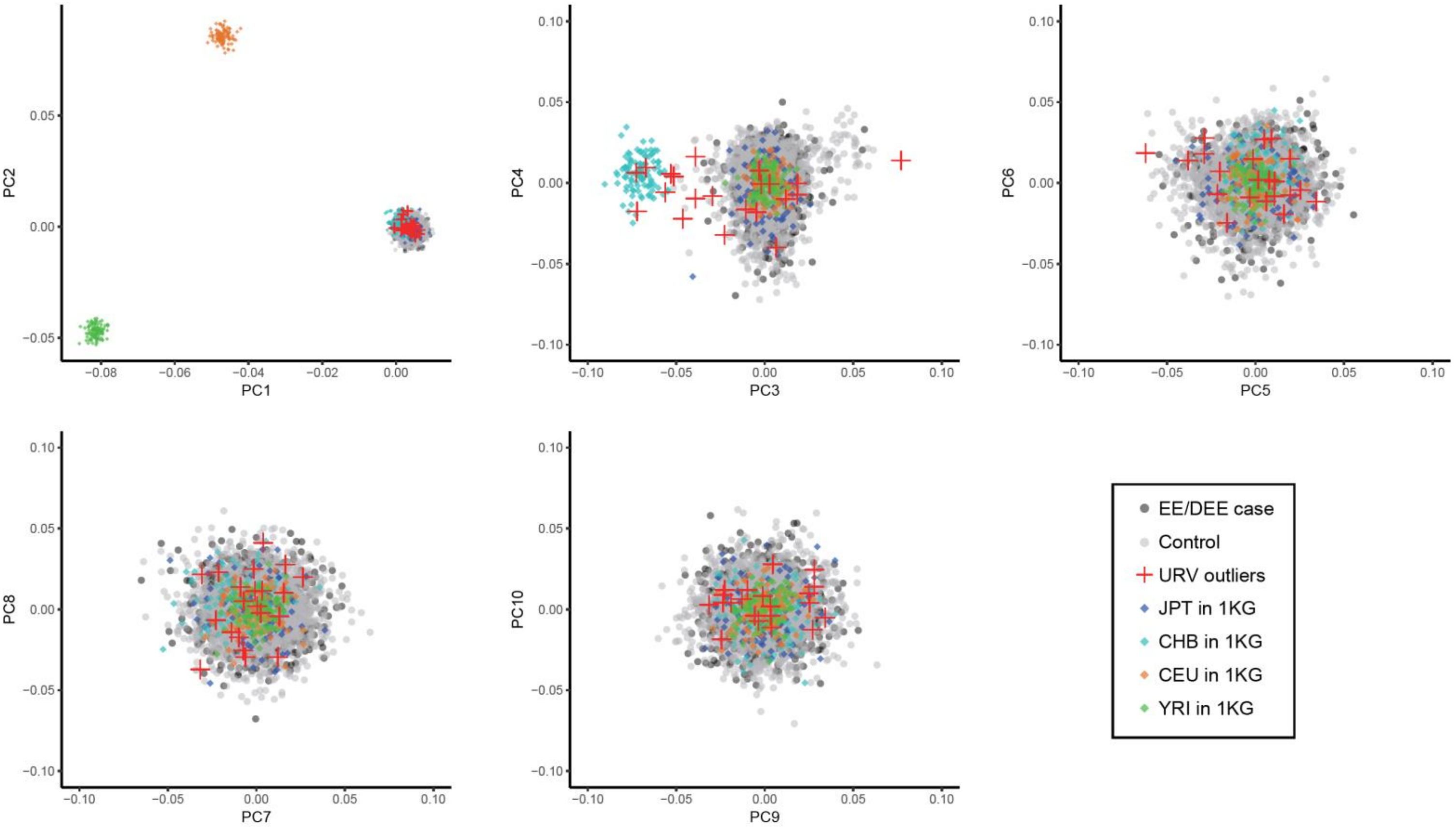

Supplementary Figure 2. Principal component analysis

Plots of the first ten (each two in a graph) principal components (PC) estimated from the genotype data of 3,130 individuals (749 EE/DEE cases [black circles] and 2,381 controls [gray circles]) originally included in this study, and four populations (JPT: Japanese in Tokyo [blue diamonds], CHB: Han Chinese in Beijing [cyan diamonds], CEU: Utah residents with Northern and Western European ancestry from the CEPH collection [orange diamonds], and YRI: Yoruba in Ibadan, Nigeria [green diamonds]) from the 1000 Genomes Project[1]. 21 individuals with exceptionally small or large numbers of URVs (URV outliers) excluded from our main analyses are indicated by the red crosses. We found that some URV outliers are included in the cluster of CHB (see the figure for PC3/4), justifying exclusion of these individuals from our main analyses. After excluding URV outliers, no individual in our case-control cohort were included in the cluster of CHB.

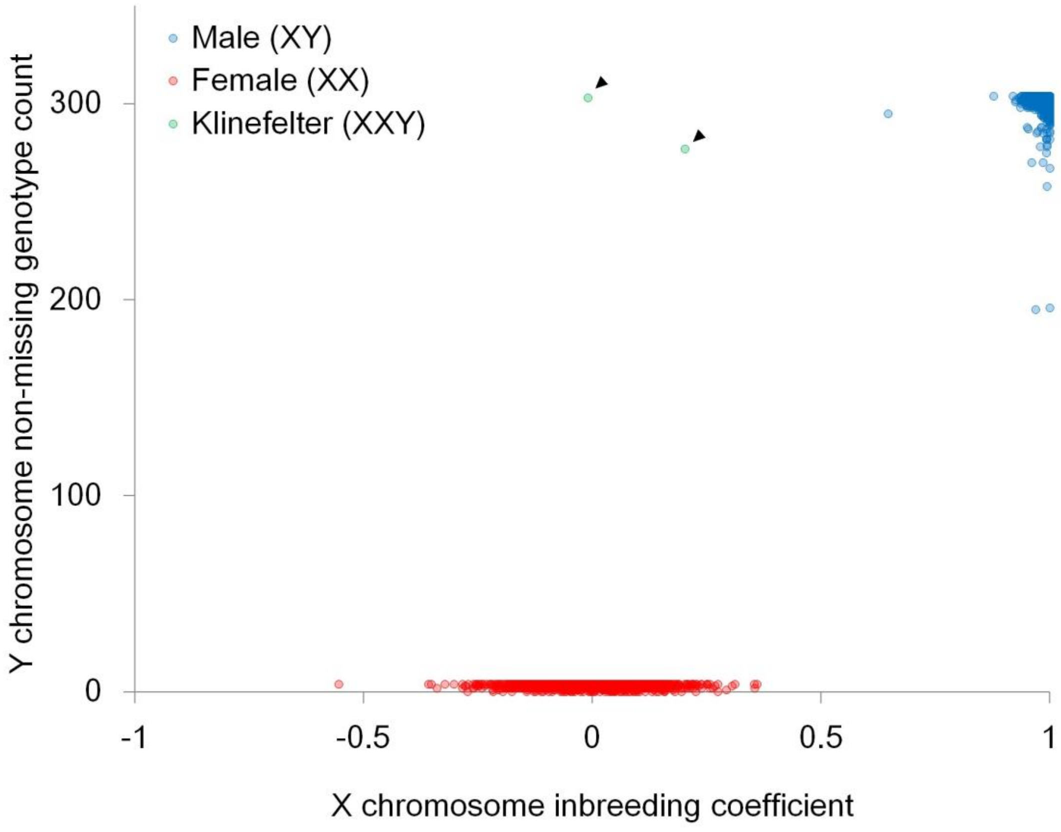

Supplementary Figure 3. Confirmation of sex using genotype data

Plots of inbreeding coefficient calculated with the X chromosome genotype data (X-axis, expected to be one in males) and the counts of non-missing genotypes on Y chromosome (Y-axis, expected to be zero in females). Through this analysis, we identified two EE/DEE individuals inferred with 47,XYY karyotype (Klinefelter syndrome), which were confirmed by their clinical records. Blue: males, red: females, green (marked with black arrowheads): Klinefelter individuals.

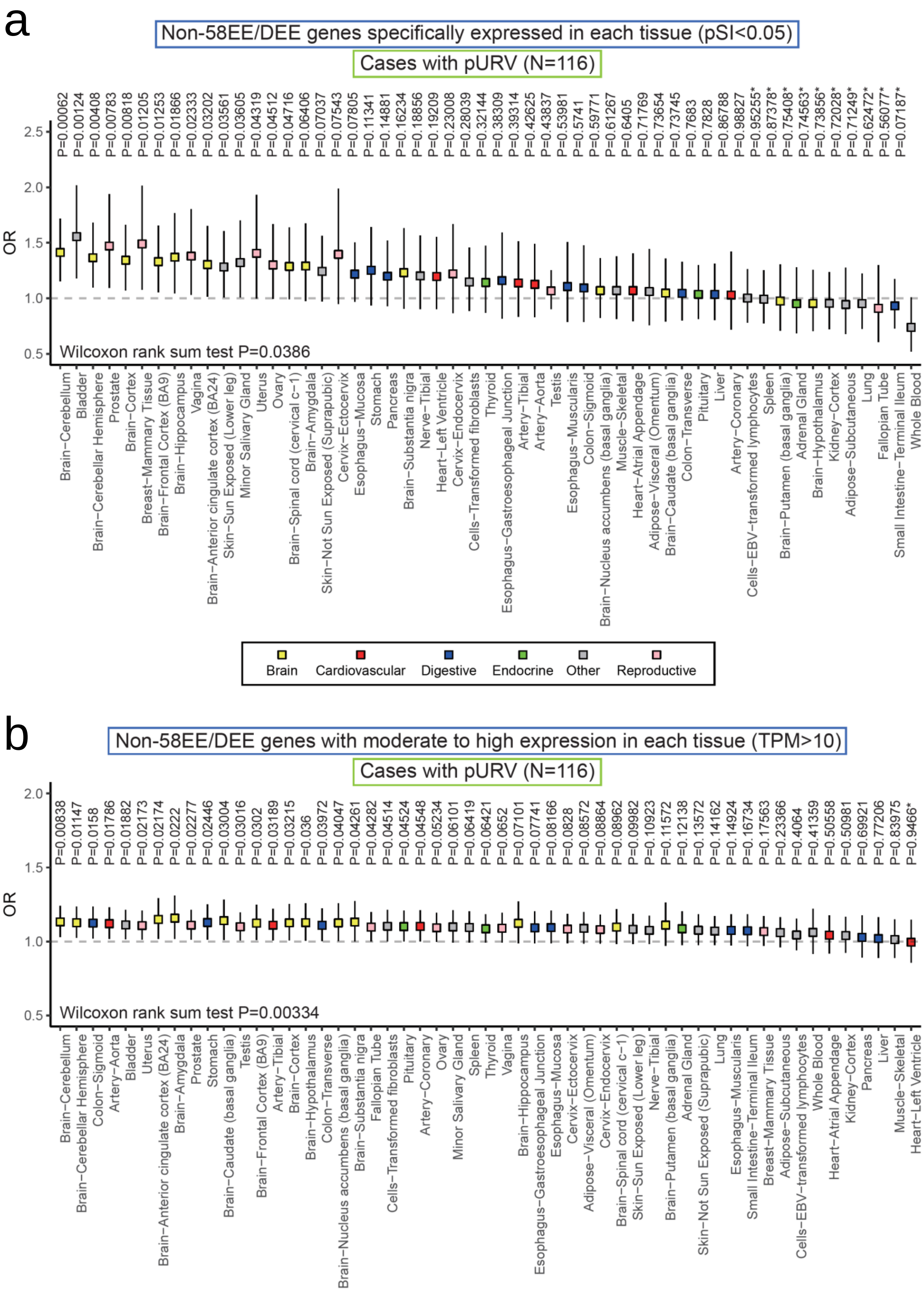
a
Non-58EE/DEE genes specifically expressed in each tissue (pSI<0.05)
Cases with pURV (N=116)
P=0.00062
P=0.00124
P=0.00408
P=0.00783
P=0.00818
P=0.01205
P=0.01253
P=0.01866
P=0.02333
P=0.03202
P=0.03561
P=0.03605
P=0.04319
P=0.04512
P=0.04716
P=0.06406
P=0.07037
P=0.07543
P=0.07805
P=0.11341
P=0.14881
P=0.16234
P=0.18856
P=0.19209
P=0.23008
P=0.28039
P=0.32144
P=0.38309
P=0.39314
P=0.42625
P=0.43837
P=0.53981
P=0.5741
P=0.59771
P=0.61267
P=0.6405
P=0.71769
P=0.73654
P=0.73745
P=0.7683
P=0.7828
P=0.86788
P=0.98827
P=0.95255*
P=0.87378*
P=0.75408*
P=0.74563*
P=0.73856*
P=0.72028*
P=0.71249*
P=0.62472*
P=0.56077*
P=0.07187*
OR
2.5
2.0
1.5
1.0
0.5
Wilcoxon rank sum test P=0.0386
Brain−Cerebellum
Bladder
Brain−Cerebellar Hemisphere
Prostate
Brain−Cortex
Breast−Mammary Tissue
Brain−Frontal Cortex (BA9)
Brain−Hippocampus
Vagina
Brain−Anterior cingulate cortex (BA24)
Skin−Sun Exposed (Lower leg)
Minor Salivary Gland
Uterus
Ovary
Brain−Spinal cord (cervical c−1)
Brain−Amygdala
Skin−Not Sun Exposed (Suprapubic)
Cervix−Ectocervix
Esophagus−Mucosa
Stomach
Pancreas
Brain−Substantia nigra
Nerve−Tibial
Heart−Left Ventricle
Cervix−Endocervix
Cells−Transformed fibroblasts
Thyroid
Esophagus−Gastroesophageal Junction
Artery−Tibial
Artery−Aorta
Testis
Esophagus−Muscularis
Colon−Sigmoid
Brain−Nucleus accumbens (basal ganglia)
Muscle−Skeletal
Heart−Atrial Appendage
Adipose−Visceral (Omentum)
Brain−Caudate (basal ganglia)
Colon−Transverse
Pituitary
Liver
Artery−Coronary
Cells−EBV−transformed lymphocytes
Spleen
Brain−Putamen (basal ganglia)
Adrenal Gland
Brain−Hypothalamus
Kidney−Cortex
Adipose−Subcutaneous
Lung
Fallopian Tube
Small Intestine−Terminal Ileum
Whole Blood
Brain
Cardiovascular
Digestive
Endocrine
Other
Reproductive
b
Non-58EE/DEE genes with moderate to high expression in each tissue (TPM>10)
Cases with pURV (N=116)
P=0.00838
P=0.01147
P=0.0158
P=0.01786
P=0.01882
P=0.02173
P=0.02174
P=0.0222
P=0.02277
P=0.02446
P=0.03004
P=0.03016
P=0.0302
P=0.03189
P=0.03215
P=0.036
P=0.03972
P=0.04047
P=0.04261
P=0.04282
P=0.04514
P=0.04524
P=0.04548
P=0.05234
P=0.06101
P=0.06419
P=0.06421
P=0.0652
P=0.07101
P=0.07741
P=0.08166
P=0.0828
P=0.08572
P=0.08864
P=0.08962
P=0.09982
P=0.10923
P=0.11572
P=0.12138
P=0.13572
P=0.14162
P=0.14924
P=0.16734
P=0.17563
P=0.23366
P=0.4064
P=0.41359
P=0.50558
P=0.50981
P=0.69921
P=0.77206
P=0.83975
P=0.9466*
OR
2.0
1.5
1.0
0.5
Wilcoxon rank sum test P=0.00334
Brain−Cerebellum
Brain−Cerebellar Hemisphere
Colon−Sigmoid
Artery−Aorta
Bladder
Uterus
Brain−Anterior cingulate cortex (BA24)
Brain−Amygdala
Prostate
Stomach
Brain−Caudate (basal ganglia)
Testis
Brain−Frontal Cortex (BA9)
Artery−Tibial
Brain−Cortex
Brain−Hypothalamus
Colon−Transverse
Brain−Nucleus accumbens (basal ganglia)
Brain−Substantia nigra
Fallopian Tube
Cells−Transformed fibroblasts
Pituitary
Artery−Coronary
Ovary
Minor Salivary Gland
Spleen
Thyroid
Vagina
Brain−Hippocampus
Esophagus−Gastroesophageal Junction
Esophagus−Mucosa
Cervix−Ectocervix
Adipose−Visceral (Omentum)
Cervix−Endocervix
Brain−Spinal cord (cervical c−1)
Skin−Sun Exposed (Lower leg)
Nerve−Tibial
Brain−Putamen (basal ganglia)
Adrenal Gland
Skin−Not Sun Exposed (Suprapubic)
Lung
Esophagus−Muscularis
Small Intestine−Terminal Ileum
Breast−Mammary Tissue
Adipose−Subcutaneous
Cells−EBV−transformed lymphocytes
Whole Blood
Heart−Atrial Appendage
Kidney−Cortex
Pancreas
Liver
Muscle−Skeletal
Heart−Left Ventricle

Supplementary Figure 4. Analysis of properties of non-58EE/DEE gene dURVs among pURV carriers using transcriptome data of 53 tissue/cell types

Results of logistic regression analysis testing association of the case-control status (116 EE/DEE cases with pURV in 58EE/DEE genes or 2,366 controls) with dURV counts in non-58EE/DEE genes that are specifically expressed (pSI: specificity index probability < 0.05)[2, 3] in each of the 53 tissue/cell types in GTEx[4] (**a**) or dURV counts in genes with moderate to high expression (TPM: transcripts per million reads > 10) in each GTEx tissue (**b**) are shown. Squares indicate odds ratios (ORs) for one additional dURV, which are color-coded by the system-level annotation as follows: brain, yellow; cardiovascular, red; digestive, blue; endocrine, green; reproductive, pink; others, gray. Bars indicate 95% CI. As in **Figs. 1** and **2** in the Main Text, genes of interest and the case subset contrasted with the controls are shown in the blue and light green boxes, respectively. Results were sorted in the order of P values obtained from logistic regression analysis. For tissues where non-58EE/DEE gene dURVs were depleted among the 116 pURV carriers (i.e. OR < 1, marked with asterisks), P values were inversely sorted. The sorted ranks of logistic regression P values for 13 brain regions and the other 40 tissues were evaluated by two-tailed Wilcoxon rank sum test, and the resulting P values are shown at the lower left corner of the graphs (we observed that brain regions were with significantly smaller logistic regression P values when compared with the other tissues).

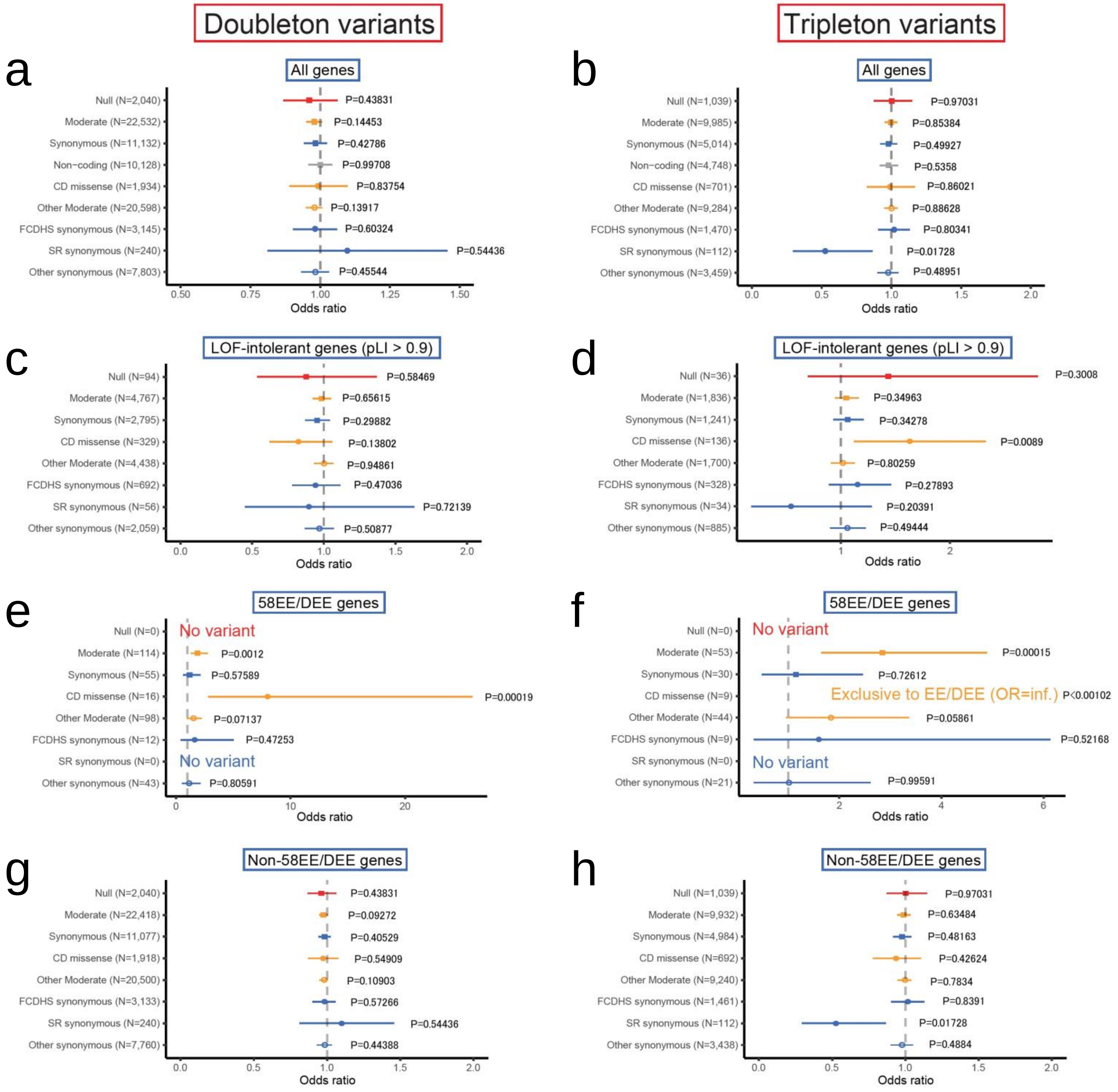

Supplementary Figure 5. Analysis of enrichment of doubleton and tripleton rare variants in EE/DEE

(**a-h**) Result of logistic regression analysis testing association between each type of doubleton/tripleton rare variants (those observed twice or three times in our overall case-control cohort and not seen in databases) and the case-control status. Left (**a**, **c**, **e**, **g**) and right (**b**, **d**, **f**, **h**) panels indicate results for doubleton and tripleton rare variants,

respectively. Odds ratios for one additional variant and 95% confidence intervals are plotted. Uncorrected P values for each test are shown beside the plots. Plots are color-coded as follows: null, red; Moderate (defined by SnpEff[5], e.g. missense and inflame), orange; synonymous, blue; non-coding, gray; and shape-coded as follows: overall functional type with no subclassification (e.g. null and Moderate), filled square; variants more likely to be functional (i.e. consensus damaging [CD] missense and frontal cortex DNase I hypersensitive site [FCDHS] and splice region [SR] synonymous), filled circle; variants less likely to be functional, open circle. The numbers of variants subjected to each analysis are indicated in the brackets. In (**a** and **b**), doubleton/tripleton rare variants in all genes were analyzed. In (**c-h**), doubleton/tripleton rare variants in the following genesets were analyzed: (**c** and **d**), LOF-intolerant genes (probability of being LOF-intolerant [pLI] > 0.9); (**e** and **f**), known 58 EE/DEE genes (58EE/DEE genes; **Supplementary Table 2**); (**g** and **h**), genes other than 58EE/DEE genes.

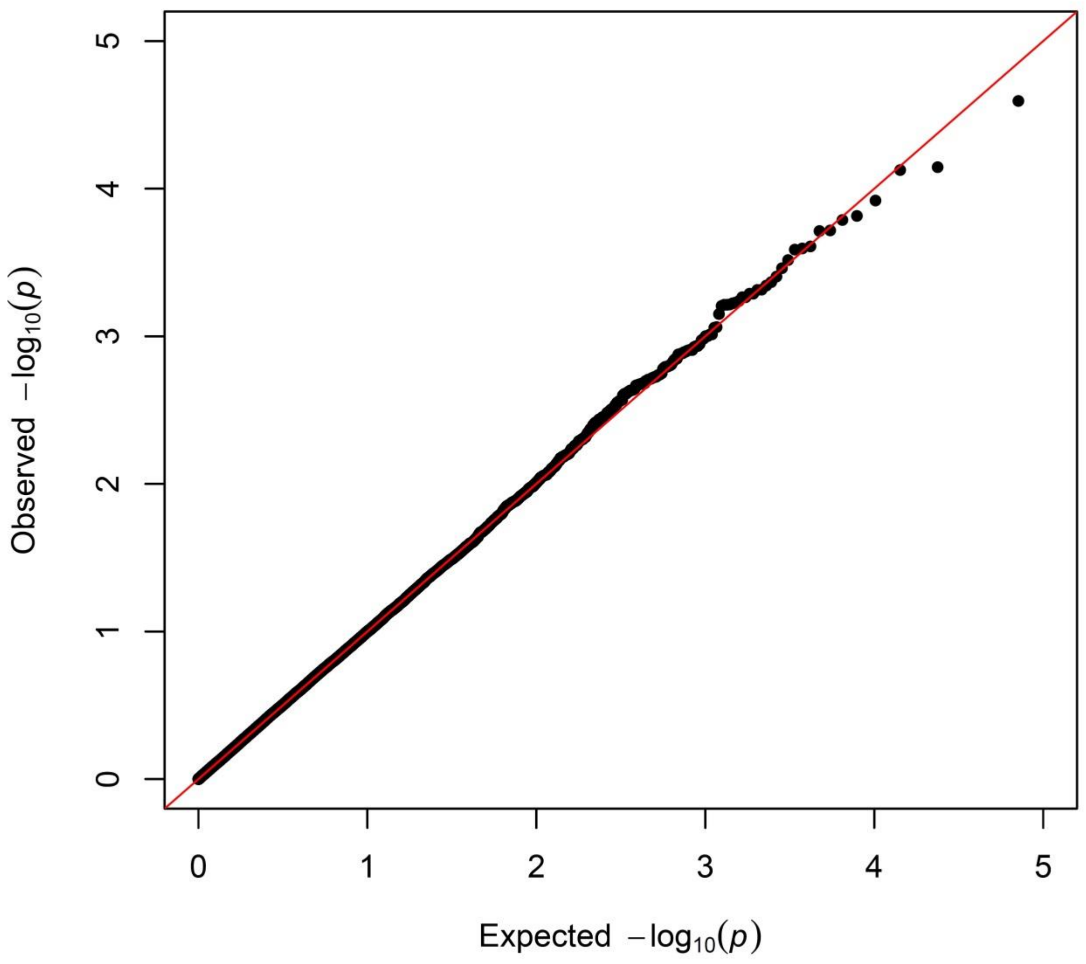

Supplementary Figure 6. Quantile-quantile plot of P values in exome-wide association study of SNPs

Expected and observed –log10 P values for 35,375 SNPs subjected to the exome-wide association study were plotted. Genomic inflation factor (lambda) was 1.003, indicating minimal population stratification in our EE/DEE case-control cohort. The red line indicates X = Y.

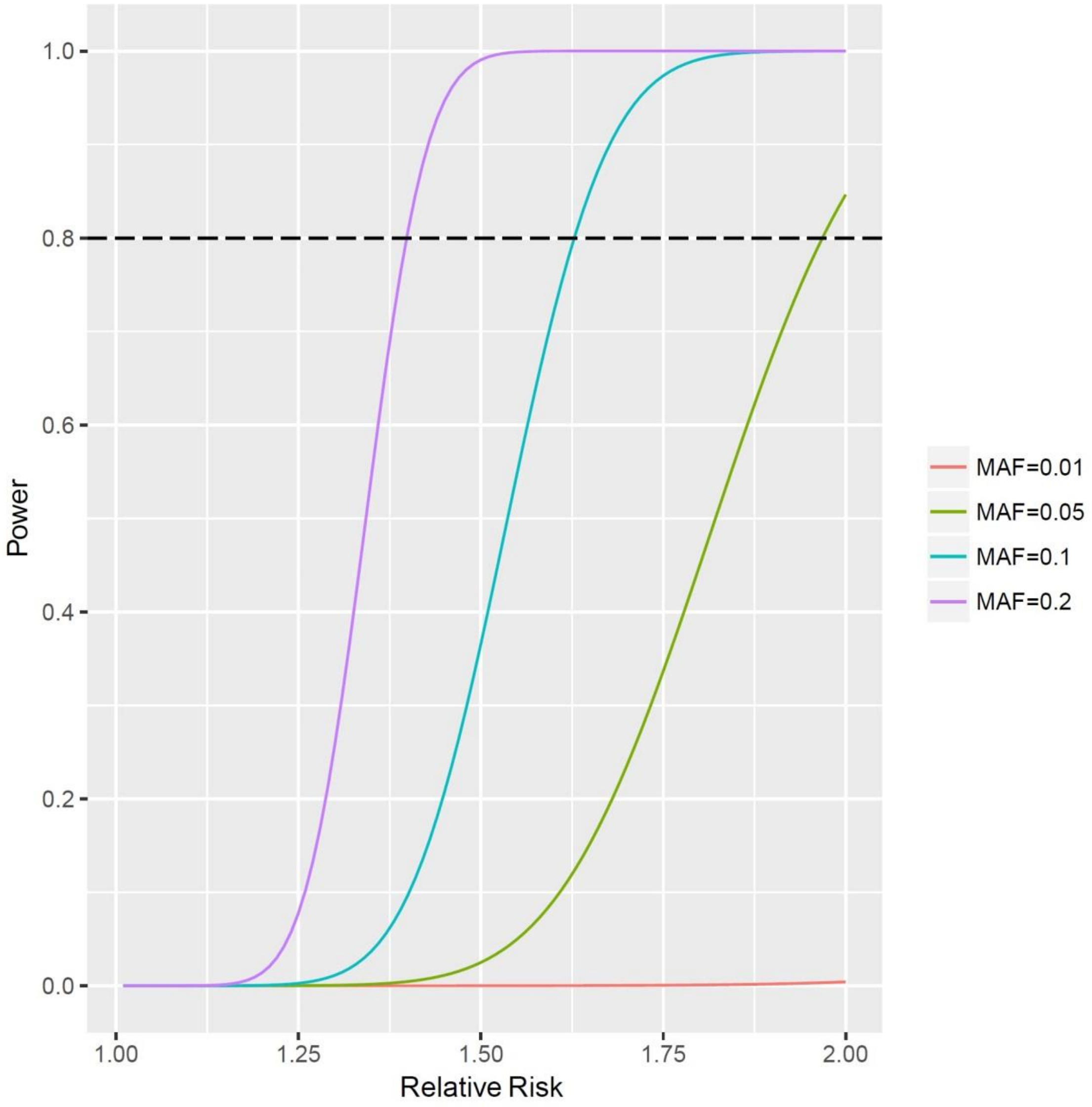

Supplementary Figure 7. Power calculation with our sample size

Plots of statistical power (Y-axis) obtained from our sample size (743 cases and 2,366 controls) to detect a SNP with the relative risk indicated in the X-axis at the level of genome-wide significance ($P = 5\times10^{-8}$). Power calculation was performed by using "pwr.2p2n.test" function in "pwr" package of R for four different minor allele frequencies (MAF): 1, 5, 10 and 20%, indicated by red, green, cyan and purple lines, respectively. The black horizontal dashed line indicates Power = 80%.

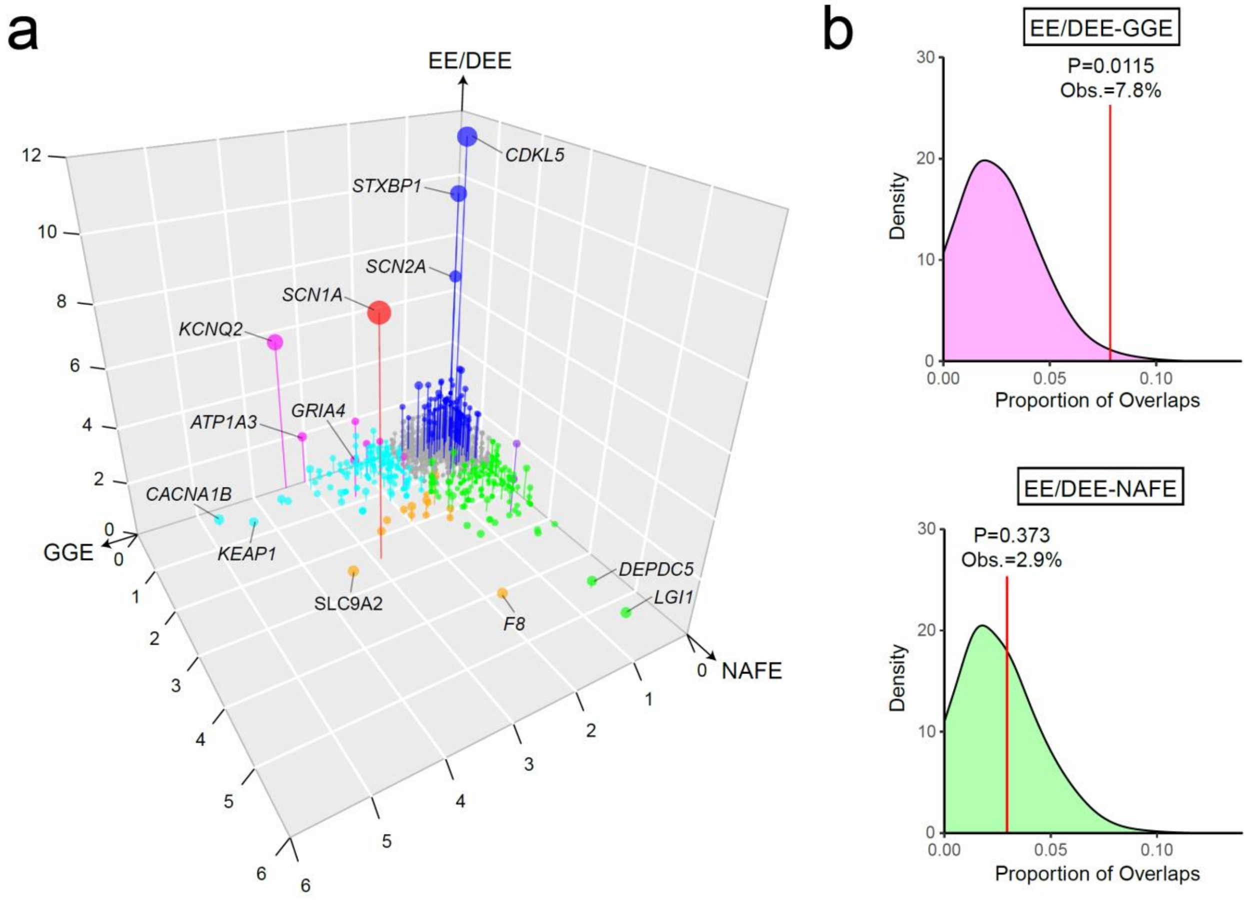

Supplementary Figure 8. Comparison between EE/DEE and common epilepsies.

(**a**) A 3D plotting of $-\log_{10}$ P values obtained from gene-based burden tests for EE/DEE (our study), genetic generalized epilepsy (GGE) and non-acquired focal epilepsy (NAFE) (a published case-control WES study by ref. [6]). Genes are color-coded as follows: red, $P < 0.05$ in EE/DEE, GGE and NAFE; magenta, $P < 0.05$ in EE/DEE and GGE; purple, $P < 0.05$ in EE/DEE and NAFE; orange, $P < 0.05$ in GGE and NAFE; blue, $P < 0.05$ in EE/DEE but not in the others; cyan, $P < 0.05$ in GGE but not in the others; green, $P < 0.05$ in NAFE but not in the others; gray, $p \geq 0.05$ in all three types of epilepsies. Point sizes represent the sum of $-\log_{10}$ P values in EE/DEE, GGE and NAFE. 13 genes with the largest sum of $-\log_{10}$ P values are labeled with their gene symbols. (**b**) Analysis of overlaps of nominally significant genes ($P < 0.05$) between EE/DEE and GGE (top) or NAFE (bottom). The observed overlaps were indicated by the red vertical lines (7.8 and 2.9% of the genes nominally significant in EE/DEE overlapped with genes nominally significant in GGE and NAFE, respectively). Expected distributions of overlaps obtained from random shuffling of the case-control labels in our cohort are shown as the light magenta (top, for analysis of GGE) and light green area (bottom, for analysis of NAFE). The corresponding P values calculated from 10,000 times of random shuffling are shown above the red vertical line.

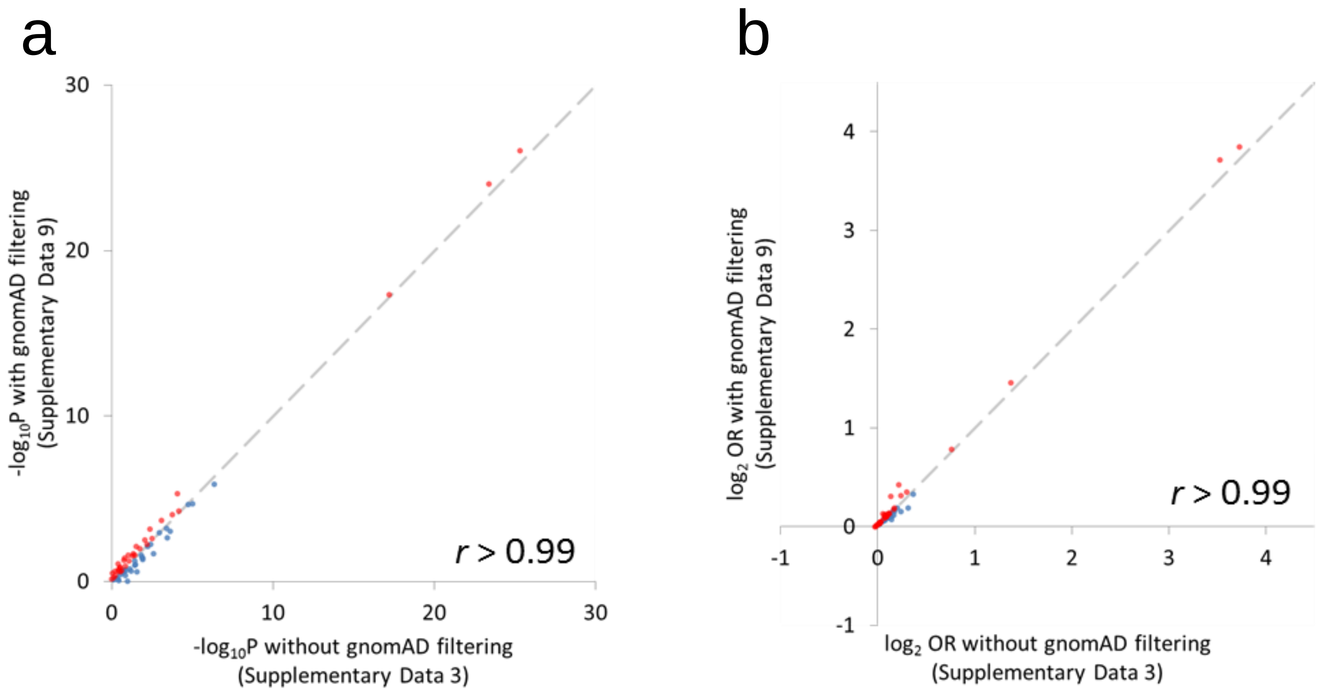

<u>Supplementary Figure 9. Plots of results in enrichment analyses of various types of URVs in EE/DEE with or without gnomAD-based filtering.</u>

(**a**) The $–\log_{10}$ P-values obtained in analyses without gnomAD-based filtering (X-axis, **Supplementary Data 3**) and those obtained in analyses with gnomAD-based filtering (Y-axis, **Supplementary Data 9**) were plotted. (**b**) The $\log_2$ ORs obtained in analyses without gnomAD-based filtering (X-axis, **Supplementary Data 3**) and those obtained in analyses with gnomAD-based filtering (Y-axis, **Supplementary Data 9**) were plotted. The red dots indicate the analyses for which a smaller (more significant) P-value or a larger OR was observed when gnomAD-based filtering was applied. The blue dots indicate the analyses for which a larger (less significant) P-value or a smaller OR was observed with gnomAD-based filtering. The gray dotted line indicates X = Y. Correlation coefficient (*r*) of X and Y is shown in each panel.

Supplementary Tables

Supplementary Table 1. Subclassification of EE/DEE cases (743 individuals that passed QCs)

Subclassification	N of individuals
Infantile spasm	268
Ohtahara syndrome	86
Migrating partial seizures of infancy	31
Dravet syndrome	24
Doose syndrome	23
Early myoclonic encephalopathy	20
Lennox-Gastaut syndrome	19
Unspecified EE/DEE	272

Supplementary Table 2. Classification of URVs

SnpEff annotation	Classification in our analysis
exon loss variant	Null
frameshift variant	Null
splice acceptor variant	Null
splice donor variant	Null
stop gained	Null
stop lost	Null
disruptive inframe deletion	Moderate
disruptive inframe insertion	Moderate
inframe deletion	Moderate
inframe insertion	Moderate
missense variant	Moderate
protein protein contact	Moderate
start lost	Moderate
initiator codon variant	Synonymous
stop retained variant	Synonymous
synonymous variant	Synonymous
3 prime UTR variant	Non-coding
5 prime UTR premature start codon gain variant	Non-coding
5 prime UTR variant	Non-coding
downstream gene variant	Non-coding
intergenic region	Non-coding
intragenic variant	Non-coding
intron variant	Non-coding
non coding exon variant	Non-coding
sequence feature	Non-coding
splice region variant	Non-coding
TF binding site variant	Non-coding
upstream gene variant	Non-coding

Supplementary Table 3. List of 58 known EE/DEE genes

Gene_Symbol	Chromosome	CodingStart(hg19)	CodingEnd	Ensembl_Canonical_Tx_ID	OMIM_ID
GNB1	1	1718769	1756892	ENST00000378609	139380
SLC2A1	1	43392711	43424322	ENST00000426263	138140
KCNA2	1	111145904	111147404	ENST00000316361	176262
HNRNPU	1	245017751	245027609	ENST00000283179	602869
ZEB2	2	145147017	145274917	ENST00000303660	605802
MBD5	2	149216327	149270510	ENST00000404807	611472
SCN2A	2	166152333	166246334	ENST00000283256	182390
SCN1A	2	166847754	166930131	ENST00000303395	182389
SLC6A1	3	11058897	11078652	ENST00000287766	137165
HCN1	5	45262022	45696195	ENST00000303230	602780
MEF2C	5	88018420	88119605	ENST00000340208	600662
PURA	5	139493766	139494735	ENST00000331327	600473
GABRA1	5	161277816	161324428	ENST00000023897	137160
GABRG2	5	161495005	161580374	ENST00000414552	137164
SYNGAP1	6	33388041	33419683	ENST00000418600	603384
KCNQ3	8	133141508	133492779	ENST00000388996	602232
STXBP1	9	130374682	130446756	ENST00000373302	602926
DNM1	9	130965749	131016993	ENST00000372923	602377
SPTAN1	9	131329019	131395613	ENST00000358161	182810
TSC1	9	135771621	135804259	ENST00000298552	605284
KCNT1	9	138594104	138684007	ENST00000298480	608167
GRIN1	9	140033938	140061979	ENST00000371546	138249
KCNC1	11	17757549	17803281	ENST00000265969	176258
SLC1A2	11	35282440	35440513	ENST00000278379	600300
GRIN2B	12	13715716	14019142	ENST00000609686	138252
SCN8A	12	52056601	52201213	ENST00000354534	600702
FOXG1	14	29236485	29237955	ENST00000313071	164874
GPHN	14	66975245	67647654	ENST00000543237	603930
UBE3A	15	25584283	25653795	ENST00000397954	601623
GABRB3	15	26792939	27184583	ENST00000541819	137192
CHD2	15	93444467	93567935	ENST00000394196	602119
TSC2	16	2098616	2138611	ENST00000219476	191092
GRIN2A	16	9857005	10274268	ENST00000330684	138253
STX1B	16	31004141	31021717	ENST00000215095	601485

GNAO1	16	56226147	56388965	ENST00000262493	139311
CACNA1A	19	13318126	13617038	ENST00000360228	601011
ATP1A3	19	42470733	42498228	ENST00000441343	182350
GRIN2D	19	48901649	48947194	ENST00000263269	602717
KCNB1	20	47989519	48099017	ENST00000371741	600397
KCNQ2	20	62037996	62103816	ENST00000354587	602235
EEF1A2	20	62119650	62129116	ENST00000217182	602959
SIK1	21	44836621	44846058	ENST00000270162	605705
PIGA	X	15339627	15350052	ENST00000333590	311770
CDKL5	X	18525216	18671664	ENST00000379989	300203
ARX	X	25022786	25033854	ENST00000379044	300382
CASK	X	41379672	41782241	ENST00000378163	300172
SYN1	X	47432262	47479127	ENST00000295987	313440
SLC35A2	X	48762003	48768913	ENST00000452555	314375
WDR45	X	48932461	48935754	ENST00000322995	300526
IQSEC2	X	53263400	53350321	ENST00000396435	300522
SMC1A	X	53407023	53449549	ENST00000322213	300040
ARHGEF9	X	62857907	62974192	ENST00000253401	300429
KIAA2022	X	73959239	73965485	ENST00000055682	300524
PCDH19	X	99551274	99663595	ENST00000373034	300460
ALG13	X	110924446	111003227	ENST00000394780	300776
SLC9A6	X	135067661	135126883	ENST00000370695	300231
SLC6A8	X	152954029	152960669	ENST00000253122	300036
MECP2	X	153295817	153363122	ENST00000453960	300005

Supplementary Table 4. Classification of pURVs in HGMD

HGMD phenotypes	Classification in our analysis
Dravet syndrome	EE/DEE
Dravet syndrome B	EE/DEE
Dravet syndrome C	EE/DEE
Encephalopathy with early epilepsy	EE/DEE
Epileptic encephalopathy	EE/DEE
Epileptic encephalopathy and development delay	EE/DEE
Epileptic encephalopathy early onset	EE/DEE
Epileptic encephalopathy early onset with involuntary movements developmental delay & intellectual disability	EE/DEE
Epileptic encephalopathy early-onset	EE/DEE
Epileptic encephalopathy infantile	EE/DEE
Epileptic encephalopathy neonatal	EE/DEE
Infantile spasms	EE/DEE
Intellectual disability & intractable epilepsy	EE/DEE
Lennox-Gastaut syndrome	EE/DEE
Migrating partial seizures of infancy	EE/DEE
Ohtahara syndrome	EE/DEE
Rett syndrome	EE/DEE
West syndrome	EE/DEE
Abnormality of the nervous system	NonEE/DEE
Alternating hemiplegia of childhood	NonEE/DEE
Alternating hemiplegia of childhood with paralysis & choreoathetosis	NonEE/DEE
Autism	NonEE/DEE
Autism spectrum disorder	NonEE/DEE
Epilepsy childhood absence with febrile seizures	NonEE/DEE
Intellectual disability	NonEE/DEE
Intellectual disability nonsyndromic	NonEE/DEE
Seizures	NonEE/DEE
Encephalopathy	Unknown/uncertain
Epilepsy infantile	Unknown/uncertain
Glucose transporter 1 deficiency syndrome	Unknown/uncertain
Neonatal epilepsy late-onset ataxia myoclonus & pain	Unknown/uncertain
Neurodevelopmental delay and hypotonia of the extremities with horizontal	Unknown/uncertain

and vertical nystagmus	
STXBP1 encephalopathy	Unknown/uncertain
Not in HGMD	Unknown/uncertain

Supplementary Table 5. List of recurrent pathogenic variants in 58EE/DEE genes

Chr	Position	Ref	Alt	Gene	Transcript_ID	cDNA_change	Protein_change	Mutation_group	N_of_cases	EE/DEE_Phenotype_in_our_cohort[a]	Phenotype_in_HGMD
9	130430439	G	A	*STXBP1*	ENST00000373302	c.875G>A	p.Arg292His	CD missense	2	Ohtahara syndrome/ Ohtahara syndrome	Infantile spasms
9	138660693	C	T	*KCNT1*	ENST00000298480	c.1420C>T	p.Arg474Cys	CD missense	2	Ohtahara syndrome/ Ohtahara syndrome	Malignant migrating partial seizures in infancy
12	52200884	C	T	*SCN8A*	ENST00000354534	c.5614C>T	p.Arg1872Trp	CD missense	2	Migrating partial seizures of infancy/ Unspecified EE/DEE	Epileptic encephalopathy infantile
20	62070004	G	A	*KCNQ2*	ENST00000354587	c.997C>T	p.Arg333Trp	CD missense	2	Ohtahara syndrome/ Ohtahara syndrome	Epilepsy benign neonatal[b]
X	18606106	C	T	*CDKL5*	ENST00000379989	c.587C>T	p.Ser196Leu	CD missense	2	Infantile spasm/ Unspecified EE/DEE	Encephalopathy with early epilepsy
12	52159459	G	A	*SCN8A*	ENST00000354534	c.2549G>A	p.Arg850Gln	CD missense	3	Migrating partial seizures of infancy/ Early myoclonic encephalopathy/ Unspecified EE/DEE	Intellectual disability and epilepsy
16	56370656	G	A	*GNAO1*	ENST00000262493	c.607G>A	p.Gly203Arg	CD missense	3	Migrating partial seizures of infancy/ Unspecified EE/DEE/ Unspecified EE/DEE	Epileptic encephalopathy
20	62126415	C	T	*EEF1A2*	ENST00000217182	c.364G>A	p.Glu122Lys	CD missense	3	Infantile spasm/ Unspecified EE/DEE/ Unspecified EE/DEE	Intellectual disability, autistic behaviours, epilepsy and characteristic facial features
9	138660694	G	A	*KCNT1*	ENST00000298480	c.1421G>A	p.Arg474His	CD missense	5	Infantile spasm/ Migrating partial seizures of infancy/ Migrating partial seizures of infancy/ Ohtahara syndrome/ Unspecified EE/DEE	Malignant migrating partial seizures in infancy

[a]Information of two or three individuals carrying the corresponding doubleton or tripleton variant is shown.

[b]According to the clinical description in the original publication (Schmitt et al., 2005), severe and global developmental delay was reported in their case with the same variant.

Supplementary Table 6. Detailed result of GO enrichment analysis

GO_category	GO_ID	GO_Name	P	BH-corrected P	Hit Count in Query List	Hit Count in Genome
GO: Biological Process	GO:0006811	ion transport	7.91E-07	2.66E-03	38	1627
GO: Biological Process	GO:0030001	metal ion transport	5.74E-06	7.67E-03	24	855
GO: Biological Process	GO:0006812	cation transport	6.84E-06	7.67E-03	28	1110
GO: Biological Process	GO:0034220	ion transmembrane transport	9.14E-06	7.69E-03	27	1064
GO: Molecular Function	GO:0015075	ion transmembrane transporter activity	9.45E-06	8.03E-03	24	873
GO: Biological Process	GO:0042592	homeostatic process	1.42E-05	9.56E-03	37	1767
GO: Biological Process	GO:0098655	cation transmembrane transport	1.88E-05	1.06E-02	21	738
GO: Biological Process	GO:0098660	inorganic ion transmembrane transport	2.59E-05	1.24E-02	21	754
GO: Biological Process	GO:0055085	transmembrane transport	3.87E-05	1.63E-02	30	1356
GO: Biological Process	GO:0098662	inorganic cation transmembrane transport	4.74E-05	1.77E-02	19	667
GO: Biological Process	GO:0070838	divalent metal ion transport	6.18E-05	1.87E-02	15	455
GO: Biological Process	GO:0006816	calcium ion transport	6.60E-05	1.87E-02	14	405
GO: Biological Process	GO:0072511	divalent inorganic cation transport	6.65E-05	1.87E-02	15	458
GO: Biological Process	GO:0048878	chemical homeostasis	7.78E-05	2.02E-02	26	1136
GO: Molecular Function	GO:0008324	cation transmembrane transporter activity	1.03E-04	2.40E-02	18	642
GO: Molecular Function	GO:0022857	transmembrane transporter activity	1.03E-04	2.40E-02	24	1014
GO: Molecular Function	GO:0046873	metal ion transmembrane transporter activity	1.24E-04	2.40E-02	14	426
GO: Molecular Function	GO:0022890	inorganic cation transmembrane transporter activity	1.41E-04	2.40E-02	16	542
GO: Biological Process	GO:0048872	homeostasis of number of cells	1.22E-04	2.94E-02	11	277
GO: Molecular Function	GO:0008509	anion transmembrane transporter activity	2.41E-04	3.42E-02	11	297
GO: Molecular Function	GO:0016874	ligase activity	3.43E-04	3.87E-02	13	415
GO: Molecular Function	GO:0072509	divalent inorganic cation transmembrane transporter activity	4.03E-04	3.87E-02	8	173
GO: Molecular Function	GO:0015085	calcium ion transmembrane transporter activity	4.10E-04	3.87E-02	7	132
GO: Molecular Function	GO:0008514	organic anion transmembrane transporter activity	5.85E-04	4.72E-02	8	183
GO: Molecular Function	GO:0031544	peptidyl-proline 3-dioxygenase activity	6.11E-04	4.72E-02	2	4
GO: Biological Process	GO:0051056	regulation of small GTPase mediated signal transduction	2.11E-04	4.75E-02	11	295
GO: Molecular Function	GO:0005215	transporter activity	6.80E-04	4.82E-02	26	1294

Supplementary Table 7. Clinical manifestations of DD/DEE cases with a confirmed de novo mutation in NF1 or CACNA1E

NF1 (ENST00000358273)

Variant	Café-au-lait spots	Neurofibroma	Lisch nodules of iris	Brain tumor	Bone abnormality	DD/ID	Infantile spasm
c.3445A>G [p.Met1149Val]	+	-	-	-	-	+ (DQ44 at 2y7m)	+
c.4835+1G>T	+	+	-	-	+	+	+
c.5330T>A [p.Val1777Asp]	-	-	-	-	-	+/- (IQ76 at 6y)	+

CACNA1E (ENST00000367573)

Variant	Seizure types (age of onset of first seizure)	EEG findings	MRI findings	Neurological features	Development	Movement disorder	Congenital contractures	Macrocephaly
c.2092T>C [p.Phe698Leu]	Epileptic spasms (3m), tonic seizures (10m)	Hypsarrhythmia, multifocal discharges	Hypoplastic corpus callosum at 3m, atrophy of corpus callosum and posterior deep white matter with high T2 signal at 2y	Spastic and dystonic quadriplegia	Profound DD: non-verbal, non-ambulatory	Dystonia	+	- (49 cm at 3y3m)
c.2104G>A [p.Ala702Thr]	Apnea (4d-present), epileptic spasms (3m), tonic seizures (3m-present)	Focal discharges (4d), hypsarrhythmia (3m), multifocal discharges (5m-present)	White matter volume loss, cortical atrophy, thin corpus callosum	Severe axial hypotonia, appendicular hypertonia	Profound DD: non-verbal, non-ambulatory	Dystonia (paroxysmal retroflexion of the neck)	+	- (52 cm at 8y)

Abbreviations: d, days; DD, developmental delay; DQ, developmental quotient; ID, intellectual disability; IQ, intelligence quotient; m, months; y, years.

Supplementary Notes

Supplementary Note 1. Detailed information of dURVs in 58EE/DEE genes in controls

When we scrutinized properties of individual null URVs in 58EE/DEE genes in controls, we found that 15/17 of these URVs are in either of the followings: 1) URVs in genes of which missense variants are known as the primary cause of EE/DEE (N = 8, in *CACNA1A*, *KCNB1*, *KCNC1*, *KCNT1*, *GRIN1* or *SIK1*), 2) URVs in isoform-specific exons (N = 3, in *GABRG2*, *MEF2C* or *TSC2*), 3) URVs in the last exon (N = 2, in *SYNGAP1* or *ZEB2*) or 4) URVs in imprinting gene (N = 2, in *UBE3A*). One of the remaining two null URVs was found in *TSC1*. Given that *TSC1* mutations on average cause milder phenotypes when compared with *TSC2*[7], the carrier may have unrecognized symptoms, or some factors contributing to resilience against the mutation[8]. The other null URV was found in *SYN1* in a female subject. Null mutations of this gene were initially identified as causal for X-linked recessive epilepsy with cognitive symptoms[9], and later potential dominant mutations in this gene were reported[10]. Therefore the heterozygous null URV of *SYN1* would be incompletely penetrant.

Regarding CD missense URVs, it would not be very surprising to observe some CD missenses in controls, because the accuracy of computational prediction is not perfect. Meanwhile, we found that three CD missense URVs are registered in HGMD (version2017.3) as "disease-causing mutation" (each one URV in *CACNA1A*, *GNAO1* and *SCN2A*). A *CACNA1A* URV (ENST00000360228:c.1594G>A [p.Glu532Lys]) is registered in HGMD, but is not linked to EE/DEE (reported in cases with episodic ataxia or benign paroxysmal torticollis of infancy). A *GNAO1* URV (ENST00000262493:c.836T>A [p.Ile279Asn]) was reported in EE/DEE as a recurrent DNM and thereby its causal role is quite convincing. We revisited the variant call for this URV and confirmed that this is likely a somatic variant (~10% of the reads support the variant allele). A *SCN2A* URV (ENST00000283256:c.1945G>A [p.Asp649Asn]) was reported in a case of Dravet syndrome but its inheritance pattern was not confirmed in the study reporting this variant.

Therefore, for most of the dURVs 58EE/DEE genes in controls, we could find some reasonable explanation why these dURVs can be observed in unaffected individuals. In addition to these dURVs in controls, we note that at least eight dURVs in 58EE/DEE genes in cases were transmitted from their clinically asymptomatic parents. These findings collectively indicate that pathogenic potentials of variants should be

carefully interpreted by utilizing various information besides simple variant classification (e.g. null, missense, synonymous etc.).

Supplementary Note 2. Consideration on possible explanations for enrichment of dURVs in non-58EE/DEE genes in EE/DEE cases carrying pURVs in 58EE/DEE genes

In the analysis of dURVs in non-58EE/DEE genes, we observed unexpected enrichment of these variants among EE/DEE cases carrying pURVs in 58EE/DEE genes, which can solely explain their EE/DEE phenotype. We carefully assessed if this observation can be explained by some factors irrelevant to the disease risk as follows:

1) Possible global genomic instability in EE/DEE cases carrying pURVs

The observed dURV enrichment in the carriers of pURVs would be explained if there is global genomic instability in these individuals, leading to generation of many germline/somatic DNMs. Under the established model of DNM rate in the human exome[11], expected per haploid per generation numbers of null, Moderate and synonymous DNMs are 0.043, 0.310 and 0.137, respectively. When there is global genomic instability in the carriers of pURVs, it is assumed that additional URVs are randomly generated as *de novo*, generally obeying these DNM rates for null, Moderate and synonymous ones (i.e. if there are additional 0.043 null DNMs on average, also there should be additional 0.310 Moderate DNMs). In our data, per-individual numbers of null, Moderate and synonymous URVs in non-58EE/DEE genes among the pURV carriers and controls were shown as in the table below.

	Per-individual null URV count	Per-individual Moderate URV count	Per-individual synonymous URV count
116 EE/DEE cases with	4.319	34.810	15.681
2,366 controls	3.774	34.770	15.251

So, there are on average additional 0.545 (4.319-3.774) null URVs in non-58EE/DEE genes in the pURV carriers. When these additional null URVs are all explained by global genomic instability, there should be additional 3.93 ($0.545 \times \frac{0.310}{0.043}$) and 1.74 ($0.545 \times \frac{0.137}{0.043}$) Moderate and synonymous URVs in these pURV carriers. However, this was not the case: there were on average 0.040 (34.810-34.770) and 0.430 (15.681-15.251) additional Moderate and Synonymous URVs in the pURV carriers. We therefore concluded that our observation cannot be fully explained by possible global genomic

instability in EE/DEE cases carrying pURVs.

2) Difference in birth years between cases and controls

In our cohort, EE/DEE cases are usually affected children and controls are adult individuals with no history of neurodevelopmental disorders. Therefore, on average cases are one to two generations younger than controls. This difference may cause increased burden of URVs in cases, because additional URVs would be generated as *de novo* during this period. However, in this case, distribution of the newly generated null, Moderate and synonymous URVs are expected to follow the ratio described above (0.043:0.310:0.137). Thus, our observation cannot be fully explained by this factor. In addition, it is known that null variants are preferentially eliminated by natural selection. Therefore, the number of additional null URVs that would be observed in a younger cohort should be smaller than what estimated from a theoretical mutation rate.

3) Other unknown/unidentifiable factors

There would be some other unknown/unidentifiable factors contributing to the observed results. However, any factors that randomly affect sequence reads and variant calls, such as random sequencing errors, are expected to generate false positives roughly following the proportion of null, Moderate impact and synonymous URVs described above (0.043:0.310:0.137). In addition, (at least some parts of) unknown/unidentifiable factors were expected to be captured as principal components that were incorporated into our regression analysis. We also note that we extensively excluded URV count outliers (**Supplementary Figure 1**), and confirmed that there was no evidence of population stratification in our cohort (**Supplementary Figure 2**). Finally, if there is a major contribution from unknown/unidentifiable factors to the observed results, it should not be expected to obtain biologically interpretable results seen in our analyses, such as enrichment of predicted functional URVs but not likely neutral URVs in EE/DEE, strong enrichment of dURVs in 58EE/DEE genes in the overall EE/DEE cohort, and re-identification of established EE/DEE genes in the burden test.

In addition to the above consideration, our *post hoc* calculation of statistical power demonstrated that the analysis of non-58EE/DEE gene dURVs among 116 EE/DEE cases carrying pURVs and 2,366 controls can achieve good statistical power when testing limited number of hypotheses (93% for $\alpha = 0.05$ and 73% for $\alpha = 0.005$). We therefore concluded that there is no good reason to suppose that the observed enrichment of non-58EE/DEE gene dURVs among the 116 EE/DEE cases carrying pURVs in 58EE/DEE

genes is a biologically meaningless finding.

Supplementary Note 3. Analysis of doubleton and tripleton rare variants

While we in this study primarily focused on singleton URVs, we also analyzed doubleton and tripleton rare variants, that is, variants observed twice and three times in our overall case-control cohort, respectively, and not seen in any of ExAC, ESP, and ToMMo databases. These groups of rare but non-private variants are expected to contain recurrent disease-causable mutations, which play an important role in EE/DEE[12]. When we analyzed doubleton/tripleton rare variants in all genes, there was no significant enrichment of any functional types of variants in EE/DEE (**Supplementary Figures 5a and 5b**). SR synonymous tripleton rare variants showed nominally significant depletion in EE/DEE (**Supplementary Figure 5b**, P = 0.0173). In the analysis focusing on LOF-intolerant genes (pLI > 0.9), there was no enrichment of any type of doubleton rare variants (**Supplementary Figure 5c**). CD missense tripleton rare variants in LOF-intolerant genes were nominally significantly enriched in EE/DEE (**Supplementary Figure 5d**, P = 0.0089). Among the 58EE/DEE genes, there was significant enrichment of CD missense doubleton and tripleton rare variants (**Supplementary Figure 5e and 5f**, P = 0.00019 and < 0.00102, respectively). There was no null doubleton or tripleton rare variant in 58EE/DEE genes, suggesting that most of these null variants in EE/DEE genes are extremely rare. When 58EE/DEE genes were excluded from the analysis, enrichment of CD missense tripleton rare variants in LOF-intolerant genes (observed in **Supplementary Figure 5d**) was no longer significant (P = 0.212). Overall, except for CD missense variants in 58EE/DEE genes, enrichment of likely functional (e.g. null and CD missense) doubleton/tripleton rare variants in EE/DEE was not as prominent as singleton URVs, in agreement with findings in recent large-scale exome sequencing studies for neuropsychiatric and neurodevelopmental disorders[13, 14].

Considering doubleton/tripleton rare variants convincingly contributing to the diagnosis of EE/DEE, there were ten doubleton (five variants each in two cases) and nine tripleton (three variants each in three cases) CD missense rare variants exclusively to the EE/DEE group (**Supplementary Table 5**). As all of these variants are previously reported mutations registered in HGMD, and confirmed as *de novo* in our cohort, they are considered as convincingly pathogenic variants. We also investigated if there are more frequent null or CD missense variants in 58EE/DEE genes exclusively found in the EE/DEE group. We identified a previously reported CD missense variant in *KCNT1* in five cases (**Supplementary Table 5**). This was the only recurrent damaging variant in 58EE/DEE genes observed four or more times in our cohort. According to the total

numbers of convincingly pathogenic singleton dURVs (n = 116, **Supplementary Data 2**) and recurrent variants (n = 24: doubleton, tripleton, and the *KCNT1* variant observed in five cases, **Supplementary Table 5**), it was indicated that extremely rare variants, such as those identified as singleton URVs in our cohort, explain a larger part of the overall genetic architecture of EE/DEE. On the other hand, we note that many of the pathogenic URVs identified as singletons in our cohort were not private events, as 49 of 116 pURVs were previously reported and registered in HGMD (**Supplementary Data 2**).

Supplementary Note 4. Exome-wide association study of SNPs

Results of analyses of URVs in EE/DEE with or without pURVs indicate existence of variants with a potential oligogenic effect. We next analyzed if exonic single nucleotide polymorphisms (SNPs: defined as variants with a minor allele frequency [MAF] > 1%) play a role in the genetic etiology of EE/DEE. By performing exome-wide association study of 35,375 SNPs, we found that there is no SNP surpassing the genome-wide significance threshold ($P < 5\times10^{-8}$) (**Supplementary Figure 6** and **Supplementary Data 5**). The smallest P value was observed for a splice-region SNP in *ADAMTS6* (rs17206779, $P = 2.55\times10^{-5}$, logistic regression), which was previously reported to be associated with osteosarcoma[15]. There was no evidence of inflation of P values (genomic inflation factor = 1.003). According to the calculation of the statistical power obtained from our sample size, it is expected that there is no common exonic SNP (MAF > 5%) changing the risk of EE/DEE twofold or more (**Supplementary Figure 7**). Proportion of the phenotypic variance explained by these exonic SNPs was not significantly larger than zero (8.2%, SE = ±7.3%, P = 0.128, estimated by GCTA-GREML[16]), while for more accurate estimation a whole-genome analysis should be required. Taken these results together, likely there is limited contribution of exonic SNPs to EE/DEE, while our sample size is insufficient to detect common SNPs with small effect sizes and low-frequency SNPs (MAF = 1-5%) with intermediate effects.

Supplementary Note 5. Comparison between EE/DEE and common epilepsies

In a recent study by the Epi4K Consortium, results of case-control rare variant analysis of common forms of epilepsy, genetic generalized epilepsy (GGE) and non-acquired focal epilepsy (NAFE), were reported[6]. Availability of this published dataset enables us to compare findings in EE/DEE and common epilepsies. By 3D-plotting the p values obtained from gene-based burden tests for EE/DEE, GGE and NAFE (combined group of familial and sporadic NAFE), we found that there are several genes that showed nominal significance (P < 0.05) across multiple categories of epilepsies (**Supplementary Figure 8a** and **Supplementary Data 7**). These genes include *SCN1A* detected in all three

categories, *KCNQ2*, *ATP1A3* and *GRIA4* observed in EE/DEE and GGE, and *SLC9A2* and *F8* found to be nominally significant in GGE and NAFE. When we assessed probability for our observation of genes overlapped between EE/DEE and GGE (8 of 102 genes nominally significant in EE/DEE: 7.8%), we found that it is unlikely to have occurred by chance (**Supplementary Figure 8b**, P = 0.0115, calculated by random shuffling of the case-control status), suggesting gene-level overlap between EE/DEE and GGE. On the other hand, overlapping genes between EE/DEE and NAFE (3 of 102 genes nominally significant in EE/DEE: 2.9%) were in the range of expectation (**Supplementary Figure 8b**, P = 0.373).

<u>*Supplementary Note 6. Confirmation of key findings by updating ExAC to gnomAD*</u>
We performed confirmatory analyses by updating ExAC to the Genome Aggregation Database (gnomAD)[17], focusing on the key findings in our study. By applying a further filtering using the gnomAD data ("non-neuro" subset of version 2.1, 104,068 exomes and 10,636 genomes), 29,841 out of the 211,988 URVs (14.1%) in 3,109 individuals were removed. In these confirmatory analyses, we also included *CACNA1E* in the list of established EE/DEE genes (so, there were "58+1EE/DEE genes" in the list for the confirmatory analyses).

When we repeated the overall enrichment analyses of various functional types of URVs in EE/DEE (in **Figs. 1** and **2**, 66 tests in total), we largely replicated the findings in analyses without the gnomAD-based filtering (**Supplementary Figure 9**, correlation coefficient > 0.99 for both $-\log_{10}$ P-values and $\log_2$ ORs in the 66 tests in **Supplementary Data 3** and **9**). We found that the additional gnomAD-based filtering led to an increase of OR in the majority of the tests (43/66, significantly larger than 0.5 at P = 0.019 when each test was treated as independent, two-tailed binomial test), indicating that the additional filtering has contributed to further concentration of disease-associated URVs. An increase of OR was clear in URV categories expected to be enriched for primary pathogenic variants, such as null or missense URVs in 58+1EE/DEE genes. On the other hand, a decrease of ORs was observed in categories likely harboring URVs contributing to EE/DEE in an oligogenic manner (e.g. non-58+1EE/DEE gene dURVs in the cases with pURVs in 58+1EE/DEE genes), may suggesting that further stringent filtering causes loss of these modifier URVs (**Supplementary Data 9**). Among the specific tests, we confirmed enrichment of dURVs in EE/DEE cases, in both 58+1EE/DEE genes (null; Bonferroni-corrected P = 1.03×10^{-16} and CD missense; Bonferroni-corrected P = 6.38×10^{-25}) and the other non-58+1EE/DEE genes (dURV; Bonferroni-corrected P = 0.00139). Enrichment of non-58+1EE/DEE gene dURVs in the subset of 118 EE/DEE

cases with pURVs in 58+1EE/DEE genes showed an OR similar to that observed before the gnomAD-based filtering (1.119 and 1.125 in the analyses with and without the filtering, respectively) with a P-value (raw-P = 0.000922) that remained significant after Benjamini-Hochberg correction (corrected-P = 0.00468) but not after Bonferroni adjustment (corrected-P = 0.0608), possibly due to loss of potential oligogenic URVs as mentioned above.

By repeating the gene-based burden test (in **Fig. 3** and **Supplementary Figure 8**) with the gnomAD-based filtering, we confirmed exome-wide significant enrichment of dURVs in five known EE/DEE genes (*CDKL5*, *STXBP1*, *SCN1A*, *SCN2A* and *KCNQ2*), while there was no gene newly reached to the significance threshold by application of the additional filtering (**Supplementary Data 10**). In general, there was little change in the analysis applying the additional gnomAD-based filtering (see "Change_in_P_vs_non-pURV_carriers" column of **Supplementary Data 10**). Regarding *NF1*, we found that all three damaging DNMs (c.3445A>G [p.Met1149Val], c.4835+1G>T and c.5330T>A [p.Val1777Asp]) were not found in the gnomAD non-neuro dataset. Therefore, exome-wide significant enrichment of damaging *NF1* DNMs in infantile spasm was unchanged. We also confirmed significant or nominally significant enrichment of dURVs in genes commonly mutated in different types of epilepsies (*SCN1A*, *KCNQ2, ATP1A3 and GRIA4*, **Supplementary Data 10**).

Supplementary Note 7. Consortium membership

The Case-control Working Group of the DEEPEN Consortium*

Atsushi Takata, M.D., Ph.D.[1], Mitsuko Nakashima, M.D., Ph.D.[1,2], Hirotomo Saitsu, M.D., Ph.D.[1,2], Takeshi Mizuguchi, M.D., Ph.D.[1], Satomi Mitsuhashi, M.D., Ph.D.[1], Yukitoshi Takahashi, M.D., Ph.D.[3], Nobuhiko Okamoto, M.D., Ph.D.[4], Hitoshi Osaka, M.D., Ph.D.[5], Kazuyuki Nakamura, M.D., Ph.D.[6], Jun Tohyama, M.D., Ph.D.[7], Kazuhiro Haginoya, M.D., Ph.D.[8], Saoko Takeshita, M.D.[9], Ichiro Kuki, M.D.[10], Tohru Okanishi, M.D., Ph.D.[11], Tomohide Goto, M.D., Ph.D.[12], Masayuki Sasaki, M.D., Ph.D.[13], Yasunari Sakai, M.D., Ph.D.[14], Noriko Miyake, M.D., Ph.D.[1], Satoko Miyatake, M.D., Ph.D.[1], Naomi Tsuchida, M.D., Ph.D.[1], Kazuhiro Iwama, M.D.[1], Gaku Minase, M.D.[1], Futoshi Sekiguchi, M.D.[1], Atsushi Fujita, Ph.D.[1], Eri Imagawa, Ph.D.[1], Eriko Koshimizu, Ph.D.[1], Yuri Uchiyama, M.D., Ph.D.[1], Kohei Hamanaka, M.D., Ph.D.[1], Chihiro Ohba, M.D., Ph.D.[1], Toshiyuki Itai, M.D.[1], Hiromi Aoi, M.D.[1], Ken Saida, M.D.[1], Tomohiro Sakaguchi, B.S[1], Kouhei Den[1], Rina Takahashi, M.S.[1], Hiroko Ikeda, M.D.[3], Tokito Yamaguchi, M.D.[3], Kazuki Tsukamoto, M.D.[3], Shinsaku Yoshitomi, M.D.[3], Taikan Oboshi, M.D.[3], Katsumi Imai, M.D.[3], Tomokazu Kimizu, M.D.[15], Yu Kobayashi, M.D.[7], Masaya Kubota, M.D., Ph.D.[16], Hirofumi Kashii, M.D.[16], Shimpei Baba, M.D.[11], Mizue Iai, M.D., Ph.D.[12], Ryutaro Kira, M.D., Ph.D.[17], Munetsugu Hara, M.D., Ph.D.[18], Masayasu Ohta, M.D.[19], Yohane Miyata, M.D., Ph.D.[20], Rie Miyata, M.D., Ph.D.[21], Jun-ichi Takanashi, M.D., Ph.D.[22], Jun Matsui, M.D.[23], Kenji Yokochi, M.D., Ph.D.[24], Masayuki Shimono, M.D., Ph.D.[25], Masano Amamoto, M.D.[26], Rumiko Takayama, M.D., Ph.D.[27], Shinichi Hirabayashi, M.D., Ph.D.[28], Kaori Aiba, M.D.[29], Hiroshi Matsumoto, M.D., Ph.D.[30], Shin Nabatame, M.D.[31], Takashi Shiihara, M.D., Ph.D.[32], Hiroo Omatsu, M.D.[3], Akito Watanabe, M.D.[3], Asako Horino, M.D.[3], Mao Fujioka, M.D.[3], Takayoshi Koike, M.D.[3], Hitoshi Ikeda, M.D.[3], Tae Ikeda, M.D.[15], Yasuhiro Suzuki, M.D., Ph.D.[15], Keiko Yanagihara, M.D., Ph.D.[15], Yukiko Mogami, M.D.[15], Kazuhiro Muramatsu, M.D., Ph.D.[5], Akihiko Miyauchi, M.D.[5], Karin Kojima, M.D., Ph.D.[5], Akira Hojo, M.D.[33], Shinichi Magara, M.D.[7], Sato Suzuki-Muromoto, M.D.[8], Takehiko Inui, M.D.[8], Yukimune Okubo, M.D.[8], Ryo Sato,

M.D., Ph.D.[8], Wakaba Endo, M.D.[8], Hikari Kaba, M.D.[9], Yoshihiro Watanabe, M.D., Ph.D.[9], Hisashi Kawawaki, M.D.[10], Go Takei, M.D.[16], Atsushi Kumagai, M.D.[16], Hiroshi Terashima, M.D.[16], Takeshi Inoue, M.D.[10], Shin Okazaki, M.D.[10], Sotaro Kanai, M.D.[11], Shinji Itamura, M.D.[11], Hirotaka Motoi, M.D.[11], Yoshihiko Saito, M.D.[13], Eri Takeshita, M.D., Ph.D.[13], Eiji Nakagawa, M.D., Ph.D.[13], Kei Iwata, M.D.[13], Kenji Sugai, M.D., Ph.D.[13], Akihiko Ishiyama, M.D., Ph.D.[13], Rie Anzai, M.D.[12], Sumimasa Yamashita, M.D., Ph.D.[12], Azusa Ikeda, M.D.[12], Yu Tsuyusaki, M.D.[12], Masaru Matsukura, M.D.[17], Ryoko Nakamura, M.D.[17], Pin Fee Chong, M.D.[17], Hiroya Nishida, M.D.[20], Shunpei Uchino, M.D.[20], Ikuko Shirai, M.D.[20], Ruri Satomi, M.D.[19], Keisuke Nakajima, M.D., Ph.D.[19], Shouichi Ohga, M.D., Ph.D.[14], Hiroyuki Torisu, M.D., Ph.D.[34], Toshiro Hara, M.D., Ph.D.[35], Seiichiro Yoshioka, M.D.[23], Atsuko Yamamoto-Arisaka, M.D.[21], Masahiro Ishii, M.D., Ph.D.[25], Koji Tominaga, M.D.[31], Yuji Inaba, M.D., Ph.D.[28], Tomohiro Chiyonobu, M.D., Ph.D.[36], Saori Tanabe, M.D., Ph.D.[37], Noriyuki Akasaka, M.D.[38], Muneaki Matsuo, M.D., Ph.D.[39], Yuji Kumagaya, M.D.[40], Shin-ichiro Hamano, M.D., Ph.D.[40], Satoru Takahashi, M.D., Ph.D.[41], Shunsuke Ogaya, M.D.[42], Keitaro Yamada, M.D., Ph.D.[42], Kyoko Takano, M.D., Ph.D.[43], Mina Yokoyama, M.D.[44], Kazuki Yamamoto, M.D.[44], Atsuro Daida, M.D.[44], Yuichi Takami, M.D., Ph.D.[45], Yuji Sugawara, M.D.[46], Hideki Hoshino, M.D., Ph.D.[47], Gaku Yamanaka, M.D., Ph.D.[48], Masahiro Ito, M.D.[49], Takashi Ichiyama, M.D., Ph.D.[50], Naoko Ishihara, M.D., Ph.D.[51], Hisako Ishiwata, M.D.[52], Kentaro Shirai, M.D.[53], Masafumi Morimoto, M.D., Ph.D.[54], Kazuo Okanari, M.D.[55], Ayako Hattori, M.D., Ph.D.[56], Misaki Nakashima, M.D.[55], Daisuke Ieda, M.D.[56], Shinji Saitoh, M.D., Ph.D.[56], Satoru Kobayashi, M.D., Ph.D.[57], Yonehiro Kanemura, M.D., Ph.D.[58], Katsuhiro Kobayashi, M.D.[59], Tetsuhiro Fukuyama, M.D.[60], Yusuke Aoki, M.D.[61], Hisayuki Maeda, M.D.[62], Yuji Fujii, M.D.[63], Hiroaki Ono, M.D.[64], Nobuko Moriyama, M.D.[65], Akira Kumakura, M.D.[66], Hiroshi Arai, M.D.[67], Shinjiro Akaboshi, M.D., Ph.D.[68], Masahiko Hiyane, M.D.[69], Masami Togawa, M.D.[70], Takeshi Matsushige, M.D., Ph.D.[71], Hiroko Baber Matsushita, M.D., Ph.D.[72], Tatsuharu Sato, M.D.[73], Kazuo Kodama, M.D.[74], Yoshihiro Maegaki, M.D., Ph.D.[75], Manabu Tanaka, M.D.[76], Shuei Watanabe, M.D.[77], Kuriko Kagitani-Shimono, M.D., Ph.D.[78], Atsushi Sato, M.D., Ph.D.[79], Naka Saito, M.D., Ph.D.[80], Kengo Moriyama, M.D.[81], Ayako Kashimada, M.D.[81], Yoshinobu Oyazato, M.D., Ph.D.[82], Shinobu Fukumura, M.D., Ph.D.[83], Mitsugu Uematsu, M.D.[84], Hirofumi Inoue, M.D., Ph.D.[71], Tadashi Shiohama, M.D., Ph.D.[74], Miyuki Toyono, M.D.[85], Ryuta Tanaka, M.D.[86], Takahito Wada, M.D., Ph.D.[87], Jun Miyahara, M.D.[88], Hidee Arai, M.D.[89], Ayako Umemura, M.D.[90], Tomoe Shinagawa, M.D.[91], Masayo Kanai, M.D., Ph.D.[92], Hiroyuki Wakamoto, M.D., Ph.D.[93], Yukihiko Konishi, M.D.[94], Yasuhiko Ago, M.D.[95], Nobutsune Ishikawa, M.D., Ph.D.[96], Manami Akasaka, M.D., Ph.D.[97], Tomoya Takeuchi, M.D., Ph.D.[98], Nobusuke Kimura, M.D.[99], Tsunehiko Kurokami, M.D.[100], Takafumi Higashiguchi, M.D.[101], Chikako Ogawa, M.D., Ph.D.[102], Takafumi Sakakibara, M.D., Ph.D.[103], Yuichi Abe, M.D., Ph.D.[104], Makiko Kaga, M.D., Ph.D.[105], Hirokazu Oguni, M.D., Ph.D.[106], Takashi Enokizono, M.D.[107], Kenji Ida, M.D., Ph.D.[108], Shigeru Kimura, M.D., Ph.D.[109], Naoki Ando, M.D., Ph.D.[110], Kazue Kimura, M.D., Ph.D.[111], Elina Taniguchi, M.D.[112], Shigehiro Nagai, M.D.[113], Charles Marques Lourenco, M.D., Ph.D.[114], Takahiro Yamamoto, M.D., Ph.D.[115], Hirokazu Kurahashi, M.D., Ph.D.[116], Yuji Hashimoto, M.D.[117], Hiroshi Suzumura, M.D., Ph.D.[118], Yoshinori Kobayashi, M.D.[119], Mutsumi Sato, M.D.[120], Takeshi Tsuji, M.D., Ph.D.[121], Tsuyoshi Omi, M.D.[122], Fumihito Nozaki, M.D.[123], Mariko Ikegami, M.D.[124], Yoshio Makita, M.D., Ph.D.[125], Kazushi Miya, M.D., Ph.D.[126], Mari Matsuo, M.D., Ph.D.[127], Takeshi Kumagai, M.D., Ph.D.[128], Mitsuhiro Kato, M.D., Ph.D.[6,33] & Naomichi Matsumoto, M.D., Ph.D.[1]

***The DEEPEN (DEvelopmental and EPileptic ENcephalopathy) Consortium is a framework for large-scale collaboration to *deepen* our understanding of developmental and epileptic encephalopathy.**

1 Department of Human Genetics, Yokohama City University Graduate School of Medicine, 3-9 Fukuura, Kanazawa-ku, Yokohama 236-0004, Japan

2 Department of Biochemistry, Hamamatsu University School of Medicine, 1-20-1 Handayama, Higashi-ku, Hamamatsu 431-3192, Japan

3 National Epilepsy Center, NHO Shizuoka Institute of Epilepsy and Neurological Disorders, 886 Urushiyama, Aoi-ku, Shizuoka 420-8688, Japan

4 Department of Medical Genetics, Osaka Women's and Children's Hospital, 840 Murodo-cho, Izumi, Osaka 594-1101, Japan

5 Department of Pediatrics, Jichi Medical University, 3311-1 Yakushiji, Shimotsuke 329-0498, Japan

6 Department of Pediatrics, Yamagata University Faculty of Medicine, 2-2-2 Iidanishi Yamagata 990-9585, Japan

7 Department of Child Neurology, Nishi-Niigata Chuo National Hospital, 1-14-1 Masago, Nishi-ku, Niigata 950-2085, Japan
8 Department of Pediatric Neurology, Miyagi Children's Hospital, 4-3-17 Ochiai, Aoba-ku, Sendai 989-3126, Japan
9 Department of Pediatrics, Yokohama City University Medical Center, 4-57 Urafunecho, Minami-ku, Yokohama 232-0024, Japan
10 Department of Pediatric Neurology, Osaka City General Hospital, 2-13-22 Miyakojimahondori, Miyakojima-ku Osaka 534-0021, Japan
11 Department of Child Neurology, Comprehensive Epilepsy Center, Seirei Hamamatsu General Hospital, 2-12-12 Sumiyoshi, Naka-ku, Hamamatsu 430-8558, Japan
12 Division of Neurology, Kanagawa Children's Medical Center, 2-138-4 Mutsugawa, Minami-ku, Yokohama 232-8555, Japan
13 Department of Child Neurology, National Center of Neurology and Psychiatry, 4-1-1 Ogawahigashi, Kodaira 187-8551, Japan
14 Department of Pediatrics, Graduate School of Medical Sciences, Kyushu University, 3-1-1 Maidashi, Higashi-ku, Fukuoka 812-8582, Japan
15 Department of Pediatric Neurology, Osaka Women's and Children's Hospital, 840 Murodo-cho, Izumi, Osaka 594-1101, Japan
16 Division of Neurology, National Center for Child Health and Development, 2-10-1 Okura, Setagaya-ku, Tokyo 157-8535, Japan
17 Department of Pediatric Neurology, Fukuoka Children's Hospital, 5-1-1 Kashiiteriha, Higashi-ku, Fukuoka 813-0017, Japan
18 Department of Pediatrics and Child Health, Kurume University School of Medicine, 67 Asahi-machi, Kurume, Fukuoka 830-0011, Japan
19 Department of Pediatrics, JA Toride Medical Center, 2-1-1 Hongo, Toride 302-0022, Japan
20 Department of Neuropediatrics, Tokyo Metropolitan Neurological Hospital, 2-6-1 Musashidai, Fuchu 183-0042, Japan
21 Department of Pediatrics, Tokyo-kita Medical Center, 4-17-56 Akabanedai, Kita-ku, Tokyo 115-0053, Japan
22 Department of Pediatrics, Tokyo Women's Medical University Yachiyo Medical Center, 477-96 Owadashinden, Yachiyo, 276-8524, Japan
23 Department of Pediatrics, Shiga University of Medical Science, Setatsukinowacho, Otsu 520-2192, Japan
24 Department of Pediatric Neurology, Seirei-Mikatahara General Hospital, 3458 Mikatahara-cho, Kita-ku, Hamamatsu 433-8558, Japan
25 Department of Pediatrics, School of Medicine, University of Occupational and Environmental Health, 1-1 Iseigaoka, Yahatanishi-ku, Kitakyushu 807-8555, Japan
26 Kutakyushu Municipal Yahata Hospital Pediatric Emergency Center, 4-18-1 Nishihonmachi, Yahatahigashi-ku, Kutakyushu 805-8534, Japan
27 Hokkaido Medical Center for Child Health and Rehabilitation, 1-240-6 Kanayama 1-jo, Teine-ku, Sapporo 006-0041, Japan
28 Division of Neurology, Nagano Children's Hospital, 3100 Toyoshina, Azumino 399-8288, Japan
29 Department o Pediatrics, Toyohashi Municipal Hospital, 50 Aza Hachiken Nishi, Aotake-Cho, Toyohashi 441-8570, Japan
30 Department of Pediatrics, National Defense Medical College, 3-2 Namiki, Tokorozawa 359-8513, Japan
31 Department of Pediatrics, Graduate School of Medicine, Osaka University, 2-2 Yamadaoka, Suita 565-0871, Japan
32 Department of Neurology, Gunma Children's Medical Center, 779 Shimohakoda, Hokkitsu-machi, Shibukawa 377-8577, Japan
33 Department of Pediatrics, Showa University School of Medicine, 1-5-8 Hatanodai, Shinagawa-ku, Tokyo 142-8666, Japan
34 Present address: Section of Pediatrics, Department of Medicine, Fukuoka Dental College, 2-15-1 Tamura, Sawara-ku, Fukuoka 814-0175, Japan

35 Present address: Fukuoka Children's Hospital, 5-1-1 Kashiiteriha, Higashi-ku, Fukuoka 813-0017, Japan
36 Department of Pediatrics, Kyoto Prefectural University of Medicine, 465 Kajii-cho, Kamigyo-ku, Kyoto 602-8566, Japan
37 Department of Pediatrics, Nihonkai General Hospital, 30 Akiho-cho, Sakata 998-8501, Japan
38 Department of Pediatrics, Nishi-Niigata Chuo National Hospital, 1-14-1 Masago, Nishi-ku, Niigata 950-2085, Japan
39 Department of Pediatrics, Saga University, Faculty of Medicine, 5-1-1 Nabeshima, Saga 849-8501, Japan
40 Division of Neurology, Saitama Children's Medical Center, 1-2 Shintoshin, Chuou-ku, Saitama 330-8777, Japan
41 Department of Pediatrics, Asahikawa Medical University, 2-1-1-1 Midorigaoka-Higashi, Asahikawa 078-8510, Japan
42 Department of Pediatric Neurology, Aichi Prefectural Colony Central Hospital, Aichi Human Service Center, 713-8 Kamiya-cho, Kasukai, 480-0392, Japan
43 Department of Medical Genetics, Shinshu University School of Medicine, 3-1-1 Asahi, Matsumoto 390-8621, Japan
44 Department of Pediatrics, St. Luke's International Hospital, 9-1 Akashi-Cho, Chuo-ku, Tokyo 104-8560, Japan
45 Department of Pediatrics, Japanese Red Cross Society Himeji Hospital, 1-12-1 Shimoteno, Himeji 670-8540, Japan
46 Department of Pediatrics, Soka Municipal Hospital, 2-21-1, Soka, Soka, Saitama, 340-8560, Japan
47 Department of Pediatrics, Teikyo University, School of Medicine, 2-11-1 Kaga, Itabashi-ku, Tokyo 173-8606, Japan
48 Department of Pediatrics, Tokyo Medical University, 6-7-1 Nishi-shinjuku, Shinjuku-ku, Tokyo, 160-0023, Japan
49 Department of Pediatrics, Tokyo Metropolitan Bokutoh Hospital, 4-23-15 Kotobashi, Sumida-ku, Tokyo 130-8575, Japan
50 Department of Pediatrics, Tsudumigaura Medical Center for Chidren with Disabilities, 752-4 Kume, Shunan 745-0801, Japan
51 Department of Pediatrics, Fujita Health University School of Medicine, 1-98 Dengakugakubo, Kutsukake-cho, Toyoake 470-1192, Japan
52 Home Care Clinic for Children Aozora Sumida, Marna Bld.2F, Higashikomagata 1-3-15, Sumida-ku, Tokyo, 130-0005, Japan
53 Department of Pediatrics, Tsuchiura Kyodo Hospital, 4-1-1 Ohtsuno, Tsuchiura 300-0028, Japan
54 Department of Medical Science, School of Nursing, Kyoto Prefectural University of Medicine, Kawaramachi-Hirokoji, Kamigyo-ku, Kyoto 602-8566, Japan
55 Department of Pediatrics, Oita University Faculty of Medicine, 1-1 Idaigaoka, Hasama, Yufu 879-5593, Japan
56 Department of Pediatrics and Neonatology, Nagoya City University Graduate School of Medical Sciences, 1 Kawasumi, Kawasumi-cho, Mizuho-ku, Nagoya 467-8601, Japan
57 Department of Pediatrics, Nagoya City West Medical Center, 1-1-1 Hirate-cho, Kita-ku, Nagoya 462-8508, Japan
58 Department of Biomedical Research and Innovation, Institute for Clinical Research, Osaka National Hospital, National Hospital Organization, 2-1-14 Hoenzaka, Chuo-ku, Osaka 540-0006, Japan
59 Department of Child Neurology, Okayama University Graduate School of Medicine, Dentistry and Pharmaceutical Sciences, 5-1 Shikatacho 2-chome, Kita-ku, Okayama 700-8558, Japan
60 Department of Neuropediatrics, Nagano Children's Hospital, 3100 Toyoshina, Azumino 399-8288, Japan
61 Department of Pediatric Neurology, Aichi Children's Health and Medical Center, 7-426 Morioka-cho, Obu 474-0031, Japan
62 Department of Pediatrics, Faculty of Medicine, Saga University, 1 Honjomachi, Saga 840-8502, Japan

63 Department of Pediatrics, Hiroshima City Funairi Citizens Hospital, 14-22 Funairisaiwai-cho, Naka-ku, Hiroshima, 730-0844, Japan
64 Department of Pediatrics, Hiroshima Prefectural Hospital, 1-5-54 Ujina Kanda, Minami-ku, Hiroshima 734-0844, Japan
65 Department of Pediatrics, Hitachi.Ltd., Hitachinaka General Hospital, 20-1 Ishikawa-cho, Hitachinaka 312-0057, Japan
66 Department of Pediatrics, Kitano Hospital, 2-4-20 Ogimachi, Osaka, Kita-ku, Osaka 530-8480, Japan
67 Department of Pediatrics, Morinomiya Hospital, 2-1-88 Morinomiya, Jouto-ku, Osaka 536-0025, Japan
68 Department of Pediatrics, National Hospital Oganization Tottori Medical Center, 876 Mitsu, Tottori 689-0203, Japan
69 Department of Pediatrics, Okinawa Prefectural Southern Medical Center children's Medical Center, 118-1 Arakawa, Shimajirigun, Haebaru-cho, Okinawa 901-1193, Japan
70 Department of Pediatrics, Tottori prefectural central hospital, 730 Ezu, Tottori 680-0901, Japan
71 Department of Pediatrics, Yamaguchi University Graduate School of Medicine and Health Sciences, 1-1-1 Minamikogushi, Ube, Yamaguchi 755-8505, Japan
72 Department of Pediatrics, Kyoto City Hospital, 1-2 Takada, Mibu Higashi, Nakagyo-ku, Kyoto 604-8845, Japan
73 Depertment of Pediatrics, Nagasaki University Hospital, 1-7-1 Sakamoto, Nagasaki 852-8588, Japan
74 Division of Child Neurology, Chiba Children's Hospital, 579-1 Hetacho, Midori-ku, Chiba 266-0007, Japan
75 Division of Child Neurology, Institute of Neurological Sciences, Faculty of Medicine, Tottori Univesity, 36-1 Nishi-cho, Yonago 683-8504, Japan., Japan
76 Division of General Pediatrics, Saitama Children's Medical Center, 1-2 Shintoshin, Chuou-ku, Saitama 330-8777, Japan
77 Division of Neurology, Miyagi Children's Hospital, 4-3-17 Ochiai, Aoba-ku, Sendai 989-3126, Japan
78 United Graduate School of Child Development, Osaka University, 2-2 Yamadaoka, Suita 565-0871, Japan
79 Department of Pediatrics, The University of Tokyo Hospital, 7-3-1 Hongo, Bunkyo-ku, Tokyo 113-8655, Japan
80 Department of Pediatrics, Tsuruoka Municipal Shonai Hospital, 4-20 Izumimachi, Tshuruoka 997-8515, Japan
81 Department of Pediatrics, Tokyo Medical and Dental University, 1-5-45 Yushima, Bunkyo-ku, Tokyo 113-8510, Japan
82 Department of Pediatrics, Kakogawa Central City Hospital, 439 Honmachi, Kakogawa-cho, Kakogawa 675-8611, Japan
83 Department of Pediatrics, Sapporo Medical University School of Medicine, South-1, West-16, Chuo-ku, Sapporo 060-8543, Japan
84 Department of Pediatrics, Tohoku University School of Medicine, 1-1 Seiryomachi, Aobaku, Sendai 980-8574, Japan
85 Akita Prefectural Center on Development and Disability, 3-128 Suwanosawa, Kamikitade Momosaki, Akita 010-1407, Japan
86 Department of Child Health, Faculty of Medicine, University of Tsukuba, 1-1-1 Tennodai, Tsukuba 305-8575, Japan
87 Department of Medical Ethics and Medical Genetics, Kyoto University Graduate School of Medicine, Yoshida Konoe-cho, Sakyo-ku, Kyoto 606-8501, Japan
88 Department of Neonatology, Seirei Hamamatsu General Hospital, 2-12-12 Sumiyoshi, Hamamatsu 430-8558, Japan
89 Department of neuropediatrics, Yokohama Medical and Welfare Centre, Konan, 4-6-20, Konandai, Konan-ku, Yokohama 234-0054, Japan
90 Department of Pediatric Neurology, Toyota Municipal Child Development Center, 2-19

Nishiyama-cho, Toyota 471-0062, Japan
91 Department of pediatrics, Aomori National Hospital, 155-1 Hirano, Namioka Megasawa, Aomori 038-1331, Japan
92 Department of Pediatrics, Division of Neonatology, Saitama Medical Center, Saitama Medical University, 1981 Kamoda, Kawagoe 350-8550, Japan
93 Department of Pediatrics, Ehime Rehabilitation Center for Children, 2135 Tanokubo, Toon 791-0212, Japan
94 Department of Pediatrics, Faculty of Medicine, Kagawa University, 1750-1, Ikenobe, Miki-cho, Kita-gun, Kagawa761-0793, Japan
95 Department of Pediatrics, Gifu University, 1-1 Yanagido, Gifu 501-1194, Japan.
96 Department of Pediatrics, Hiroshima University Hospital, 1-2-3 Kasumi, Minami-ku, Hiroshima 734-8551, Japan
97 Department of Pediatrics, Iwate Medical University, School of Medicine, 19-1 Uchimaru, Morioka 020-8505, Japan
98 Department of Pediatrics, Japanese Red Cross Nagoya Daiichi Hospital, 3-35, Michishita-cho, Nakamura-ku, Nagoya 453-8511, Japan
99 Department of Pediatrics, Japanese Red Cross Otsu Hospital, 1-35 Nagara, Ostu 520-8511, Japan
100 Department of Pediatrics, Kawaguchi Municipal Medical Center, 180 Nishi-Araijuku, Kawaguchi 333-0833, Japan
101 Department of Pediatrics, Matsubara Tokushukai Hospital, 7-13-26 Amamihigashi, Matsubara 580-0032, Japan
102 Department of Pediatrics, Nagoya University Graduate School of Medicine, 65 Tsurumai-cho, Showa-ku, Nagoya 466-8550, Japan
103 Department of Pediatrics, Nara Medical University, 840 Shijo-Cho, Kashihara 634-8521, Japan
104 Department of Pediatrics, Saitama Medical University, 38 Morohongo, Moroyama-machi, Iruma-gun, Saitama 350-0495, Japan
105 Department of Pediatrics, Tokyo Metropolitan Tobu Medical Center for Children with Developmental Disabilities, 3-3-25, Shinsuna, Koto-ku, Tokyo, 136-0075, Japan
106 Department of Pediatrics, Tokyo Women's Medical University, 8-1 Kawada-cho, Shinjuku-ku, Tokyo 162-8666, Japan
107 Department of Pediatrics, University of Tsukuba Hospital, 2-1-1 Amakubo, Tsukuba 305-8576, Japan
108 IDA Clinic, Racto D 35 Anshu, Minamiyashiki-cho, Yamashina-ku, Kyoto 607-8011, Japan
109 Japanese Red Cross Akita College of Nursing, 17-3 Nawashirosawa, Saruta, Kamikitate, Akita 010-1493, Japan
110 Josai Kids Clinic, 4-19-10 Josai, Nishi-ku, Nagoya 451-0031, Japan
111 Segawa Memorial Neurological Clinic for Children, 2-8 Surugadai Kanda, Chiyoda-ku, Tokyo 101-0062, Japan
112 Department of Pediatrics, University of Miyazaki Hospital, 5200 Kihara, Kiyotake-cho, Miyazaki 889-1692, Japan
113 Department of Child Neurology, Shikoku Medical Center for Children and Adults, 2-1-1, Senyu Cho, Zentsuji 760-8507, Japan
114 Department of Clinical Genetics, Centro Universitario Estacio de Ribeirao Preto, Rua Abrahao Issa Halack, 980 - Ribeirania, Ribeirao Preto - SP, Brazil
115 Department of Disability Medicine, Department of Pediatrics, Graduate School of Medicine, Gifu University, 1-1 Yanagido, Gifu 501-1194, Japan.
116 Department of Pediatrics, Aichi Medical University, 1-1 Yazakokarimata, Nagakute 480-1195, Japan
117 Department of Pediatrics, Chiba Kaihin Municipal Hospital, 3-31-1 Isobe, Mihama-ku, Chiba 261-0012, Japan
118 Department of Pediatrics, Dokkyo Medical University, 880 Kita-kobayashi, Mibu-machi, Shimotsuga-gun, Tochigi 321-0293, Japan
119 Department of Pediatrics, Kobe City Medical Center General Hospital, 2-2-1,

Minatojimaminamimachi, Chuo-ku, Kobe 650-0047, Japan
120 Department of Pediatrics, Odawara City Hospital, 46 Kuno, Odawara 250-8558, Japan
121 Department of Pediatrics, Okazaki City Hospital, 3-1 Koryujicho, Okazaki 444-8553, Japan
122 Department of Pediatrics, Okinawa Seishi Ryougoen, 2-3-1 Yorimiya, Naha 902-0064, Japan
123 Department of Pediatrics, Shiga Medical Center for Children, 5-7-30 Moriyama, Moriyama 524-0022, Japan
124 Department of Pediatrics, Tokai University Hachioji Hospital, 1838 Ishikawa-machi, Hachioji 192-0032, Japan
125 Education Center, Asahikawa Medical University, 2-1-1-1 Midorigaoka-Higashi, Asahikawa 078-8510, Japan
126 Faculty of Human Development, University of Toyama, 3190 Gofuku, Toyama 930-8555, Japan
127 Institute of Medical Genetics, Tokyo Women's Medical University, 10-22 Kawadacho, Shinjuku-ku, Tokyo 162-0054, Japan
128 NICU division, Department of Perinatal medicine, Wakayama Medical University, 811-1 Kimiidera, Wakayama 641-0012, Japan

Supplementary References

1. The 1000 Genomes Project Consortium. An integrated map of genetic variation from 1,092 human genomes. *Nature* **491**, 56-65 (2012).
2. Xu X, Wells AB, O'Brien DR, Nehorai A, Dougherty JD. Cell type-specific expression analysis to identify putative cellular mechanisms for neurogenetic disorders. *J Neurosci* **34**, 1420-1431 (2014).
3. Dougherty JD, Schmidt EF, Nakajima M, Heintz N. Analytical approaches to RNA profiling data for the identification of genes enriched in specific cells. *Nucleic Acids Res* **38**, 4218-4230 (2010).
4. GTEx Consortium. Human genomics. The Genotype-Tissue Expression (GTEx) pilot analysis: multitissue gene regulation in humans. *Science* **348**, 648-660 (2015).
5. Cingolani P*, et al.* A program for annotating and predicting the effects of single nucleotide polymorphisms, SnpEff: SNPs in the genome of Drosophila melanogaster strain w1118; iso-2; iso-3. *Fly (Austin)* **6**, 80-92 (2012).
6. Epi4K Consortium, Epilepsy Phenome/Genome Project. Ultra-rare genetic variation in common epilepsies: a case-control sequencing study. *Lancet Neurol* **16**, 135-143 (2017).
7. Dabora SL*, et al.* Mutational analysis in a cohort of 224 tuberous sclerosis patients indicates increased severity of TSC2, compared with TSC1, disease in multiple organs. *Am J Hum Genet* **68**, 64-80 (2001).
8. Chen R*, et al.* Analysis of 589,306 genomes identifies individuals resilient to severe Mendelian childhood diseases. *Nat Biotechnol* **34**, 531-538 (2016).
9. Garcia CC*, et al.* Identification of a mutation in synapsin I, a synaptic vesicle protein, in a family with epilepsy. *J Med Genet* **41**, 183-186 (2004).
10. Fassio A*, et al.* SYN1 loss-of-function mutations in autism and partial epilepsy cause impaired synaptic function. *Hum Mol Genet* **20**, 2297-2307 (2011).
11. Samocha KE*, et al.* A framework for the interpretation of de novo mutation in human disease. *Nat Genet*, (2014).
12. Hamdan FF*, et al.* High Rate of Recurrent De Novo Mutations in Developmental and Epileptic Encephalopathies. *Am J Hum Genet* **101**, 664-685 (2017).
13. Genovese G*, et al.* Increased burden of ultra-rare protein-altering variants among 4,877 individuals with schizophrenia. *Nat Neurosci* **19**, 1433-1441 (2016).
14. Ganna A*, et al.* Quantifying the Impact of Rare and Ultra-rare Coding Variation across the Phenotypic Spectrum. *Am J Hum Genet* **102**, 1204-1211 (2018).
15. Savage SA*, et al.* Genome-wide association study identifies two susceptibility loci for osteosarcoma. *Nat Genet* **45**, 799-803 (2013).
16. Yang J*, et al.* Common SNPs explain a large proportion of the heritability for human height. *Nat Genet* **42**, 565-569 (2010).

17. Karczewski KJ*, et al.* Variation across 141,456 human exomes and genomes reveals the spectrum of loss-of-function intolerance across human protein-coding genes. *bioRxiv*, 531210 (2019).
