# Supplementary material for: Comprehensive analysis of coding variants highlights genetic complexity in developmental and epileptic encephalopathy
Source: Nat Commun. 2019 Jun 7;10:2506. doi: 10.1038/s41467-019-10482-9 (PMC6555845; doi:10.1038/s41467-019-10482-9)
Supplement: Supplementary file 3 — Description of Additional Supplementary Files [file 41467_2019_10482_MOESM3_ESM.docx]

**Description of Additional Supplementary Files**

**File Name: Supplementary Data 1**

**Description:** Null and CD missense URVs in 58EE/DEE genes in controls

**File Name: Supplementary Data 2**

**Description:** List of 116 pURVs in 58EE/DEE genes

**File Name: Supplementary Data 3**

**Description:** Results of enrichment analysis of various types of URVs corrected for multiple testing

**File Name: Supplementary Data 4**

**Description:** List of non-58EE/DEE gene dURVs among the 116 individuals with pURVs in 58EE/DEE genes

**File Name: Supplementary Data 5**

**Description:** Nominally significant SNPs in exome-wide association study

**File Name: Supplementary Data 6**

**Description:** Detailed result of gene-based burden test

**File Name: Supplementary Data 7**

**Description:** Detailed data of comparison among EE/DEE, GGE and NAFE

**File Name: Supplementary Data 8**

**Description:** Inheritance patterns of dURVs in selected genes

**File Name: Supplementary Data 9**

**Description:** Confirmatory enrichment analyses of various functional types of URVs in EE/DEE by updating ExAC to gnomAD

**File Name: Supplementary Data 10**

**Description:** Confirmatory gene-based burden test by updating ExAC to gnomAD
